# BBS7–SHH Signaling Activity Regulates Primary Cilia for Periodontal Homeostasis

**DOI:** 10.3389/fcell.2021.796274

**Published:** 2021-12-07

**Authors:** Pi En Chang, Shujin Li, Hyun-Yi Kim, Dong-Joon Lee, Yoon Jeong Choi, Han-Sung Jung

**Affiliations:** ^1^ Department of Orthodontics, The Institute of Craniofacial Deformity, Yonsei University College of Dentistry, Seoul, Korea; ^2^ Division in Anatomy and Developmental Biology, Department of Oral Biology, Oral Science Research Center, BK21 FOUR Project, Yonsei University College of Dentistry, Seoul, Korea; ^3^ NGeneS Inc., Ansan-si, Korea

**Keywords:** BBS7 gene, shh signaling pathway, primary cilia, periodontal homeostasis, occlusal hypofunction

## Abstract

**Objectives:** Mechanical stimuli are essential for the maintenance of periodontal ligament (PDL) homeostasis. Although there are several studies on atrophic changes in PDL due to occlusal hypofunction, the underlying mechanism is still unknown. Here, we aimed to explore the changes of gene expression in occlusal hypofunctional PDL and elucidate the related role in maintaining the PDL homeostasis.

**Methods:** To investigate the transcriptomic difference between control and hypofunctional PDL tissue from patients, RNA sequencing was performed on 34 human teeth. The atrophic changes in PDL were evaluated by histological analysis. The effect of the Bardet-Biedl syndrome 7 (*BBS7*) knockdown was evaluated by the RT-qPCR, Western blot, wound healing, and tubule formation assay.

**Results:** We detected that the expression of *BBS7* was downregulated in occlusal hypofunctional PDL through RNA sequencing. Dynamic changes, including the number of periodontal ligament cells, alignment of collagen fibers, diameter of blood vessels, appearance of primary cilia, and torturous oxytalan fibers, were observed following occlusal hypofunction. Furthermore, Sonic hedgehog signaling (Shh) activity was closely associated with *BBS7* expression in PDL cells. In addition, the cell migration and angiogenesis were also suppressed by *BBS7* knockdown *in vitro*.

**Conclusion:** We suggest that *BBS7* plays an essential role in maintaining Shh signaling activity for PDL homeostasis.

## Introduction

**FIGURE 1:**
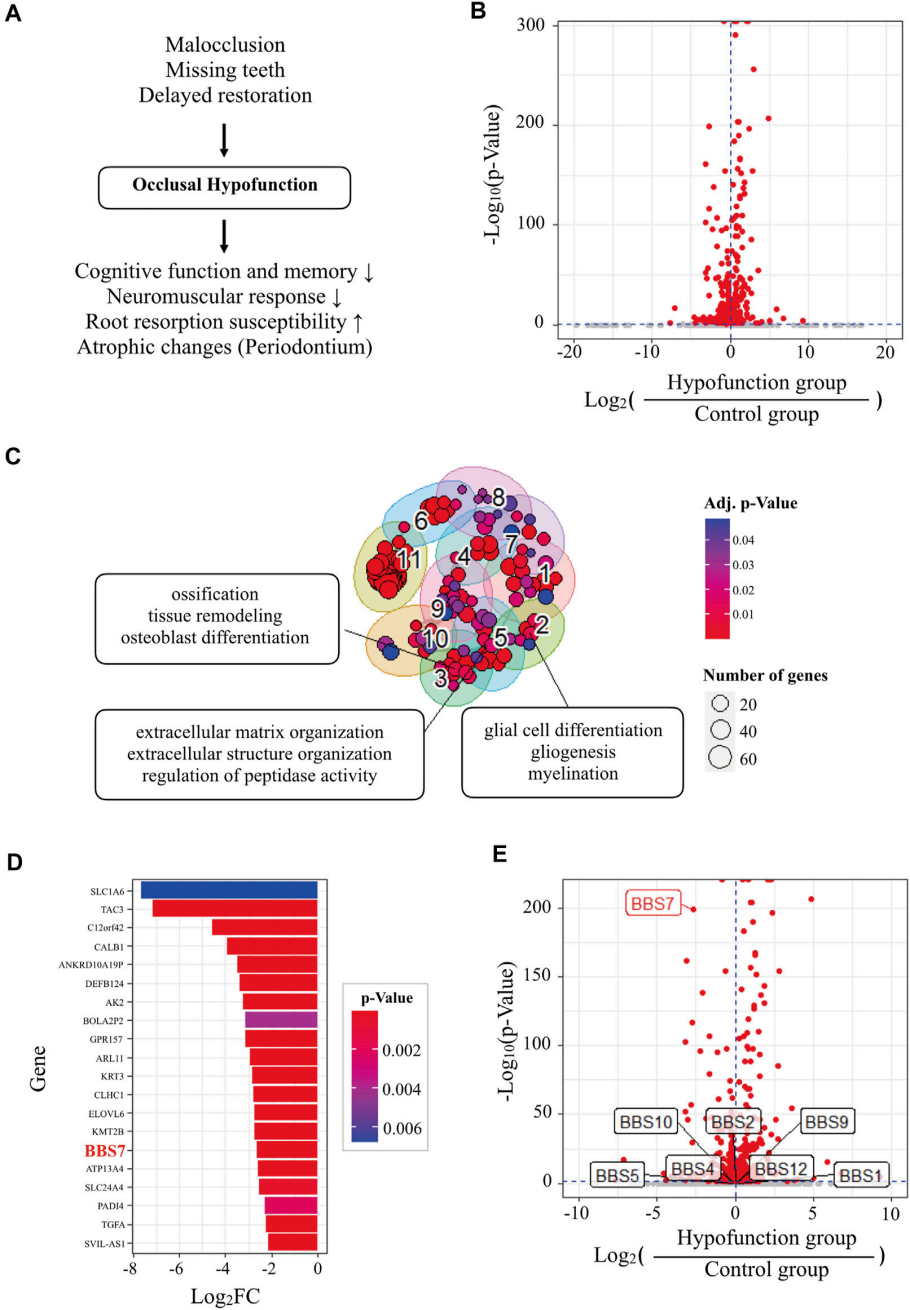
Transcriptome analysis of human PDL tissue in normal and occlusal hypofunctional tooth. **(A)** The diagram of the cause and the clinical implications of occlusal hypofunction. Lack of occlusal contact induced the occlusal hypofunction which is caused by missing and abnormal positions of teeth or other diseases. It results in various atrophic changes in the periodontium tissues, decreased neuromuscular response, and increased root susceptibility, and also affects cognitive function and memory. **(B)** Volcano plot of differentially expressed genes (DEGs) of hypofunction samples compared with control samples. Horizontal and vertical dashed blue lines indicate *p* value = 0.05 and fold change = 0, respectively. Red dots indicate significant (*p* value < 0.05) DEGs. **(C)** Gene ontology (GO) cluster map of significantly downregulated DEGs. Three top-ranked terms consisted of interesting GO clusters that were selectively labeled. Adj. *p* value = adjusted *p* value by the Benjamini-Hochberg (BH) method. **(D)** Top 20 genes of significantly downregulated DEGs. **(E)** Volcano plot of DEGs labeled with Bardet-Biedl syndrome (*BBS*) genes.

Mastication, which is an essential function of teeth, maintains homeostasis of the periodontal ligament (PDL) by delivering mechanical stimuli to the alveolar bone (Shimizu et al., 2011). It not only promotes general health but also enables cognitive function in the hippocampus (Ono et al., 2010). As occlusal force is the main source of the mechanical stimuli, loss of occlusal contact caused by malocclusion (Motokawa et al., 2013), missing teeth, and delayed restoration (Ishida et al., 2008) leads to occlusal hypofunction. Occlusal hypofunction impedes neuromuscular responses through effects on burst-generating neurons (Aung et al., 2020). Atrophic changes following occlusal hypofunction include reductions in PDL thickness (Kaneko et al., 2001), collagen fiber, and alveolar bone density (Shimizu et al., 2011; Kasahara et al., 2017), and also increase the susceptibility of root resorption (Hayashi et al., 2016) (Figure 1A). Moreover, the expression of vascular endothelial growth factor (VEGF) was also reduced in the occlusal hypofunctional PDL, which may affect periodontal remodeling and cell proliferation during wound healing (Miyagawa et al., 2009; Motokawa et al., 2015).

The PDL is a fibrous connective tissue that supports the tooth during mechanical stimulation (Hisamoto et al., 2016). Mechanical stimulation promotes PDL stem cell proliferation *via* primary cilia (Jeon et al., 2021). Primary cilia in PDL have been reported to be associated with mechanoreception (Donnelly et al., 2008), which contributes to the maintenance of the periodontium. Primary cilia are not only essential for sensing mechanics but also involved in cell signaling, such as through Sonic hedgehog (Shh) (Hampl et al., 2017). Accumulating evidence indicates that Bardet-Biedl syndrome (BBS) proteins play a crucial role in cilia function and ciliogenesis (Zaghloul and Katsanis, 2009). It has also been reported that the occlusal force can regulate the population of Gli1^+^ stem cells, which can differentiate into PDL cells, osteocytes, and cementoblasts (Men et al., 2020).

The BBSome includes seven highly conserved BBS proteins (Nachury et al., 2007). BBSome can transport membrane proteins into the cilia (Wingfield et al., 2018) and regulate Shh signaling activity (Zhang et al., 2012). Shh signaling participates in the development and tissue regeneration (Lim et al., 2018).

Although there are several studies on PDL homeostasis related to occlusal force, the underlying mechanism remains unclear. The present study aimed to explore the transcriptome alternations in occlusal hypofunctional PDL and elucidate the related role in maintaining the PDL homeostasis.

## Materials and Methods

### Patient Selection and Teeth Preparation

The study subjects were selected from patients who had visited the Department of Orthodontics between March 2018 and February 2021 and sought for orthodontic treatment. Among patients who had been scheduled for the extraction of at least two premolars, we enrolled the subjects based on the selection criteria: 1) the premolar on one side (left or right) had occlusal contact with the opposing tooth (with occlusal force; control group), while the premolar on the other side had no occlusal contact with the opposing tooth (without occlusal force; hypofunction group); 2) completion of root formation without gingival recession (less than 1 mm below the cementoenamel junction); and 3) no crown restoration or endodontic treatment. In total, 34 teeth (control group, 17; hypofunction group, 17) from ten subjects (seven males and three females) were enrolled for the present study. The study protocol was approved by the Institutional Review Board (IRB No. 2-2018-0002) of Yonsei University Dental Hospital, and written informed consent was obtained from each participant. After extraction, the middle third of the PDL was scraped and prepared for RNA sequencing (RNA-seq).

## RNA Sequencing and the Target Gene Selection

Total RNA was extracted from the middle third of the PDL using the TRIzol® reagent (#15596-026, Thermo Fisher Scientific, United States). The RNA was stored at −70°C and measured at an optical density of 260 nm. The mixtures of total RNA were incubated with Oligo dT (Gibco BRL, Rockville, NY, United States). The library was constructed and sequenced using an Illumina HiSeq 2500 Sequencer (Illumina, CA, United States). Differentially expressed genes (DEGs) between the control and hypofunction groups were identified using the R package for RNA-seq data analysis, DESeq2 (Love et al., 2014). Based on significant DEGs (adjusted *p*-value < 0.05), gene ontology (GO) analysis was performed using an R package for comparison of biological themes in gene clusters, clusterProfiler (Yu et al., 2012). The steps were followed as previously described (Kim et al., 2020).

## Cell Cultures

Human PDL was gently separated from the surface of the root and then digested in a solution of 3 mg/ml collagenase type I (Worthington Biochem, Freehold, NJ, United States) and 4 mg/ml Dispase (Roche, Mannheim, Germany) for 1 h at 37°C. Single-cell suspensions were obtained by passing the cells through a 70-µm strainer (Falcon, BD Labware, Franklin Lakes, NJ, United States). Alpha-modified Eagle’s medium (Gibco BRL, Grand Island, NY, United States) supplemented with 15% fetal bovine serum (Gibco, Life Technologies, NY, United States), 100 μmol/L ascorbic acid 2-phosphate (Gibco BRL, Grand Island, NY, United States), 2 mmol/L glutamine, and 1% pen–strep (Gibco, Life Technologies, NY, United States) was used, and the primary PDL cell was incubated at 37°C and 5% CO_2_ (Seo et al., 2004). The wound-healing assay was performed with primary PDL cells. The tubule formation assay was performed with GFP-labeled human umbilical vein endothelial cells (HUVECs, cAP-0001GFP, Angio-Proteomie, MA, United States).

To knock down Bardet-Biedl syndrome 7 (*BBS7*), shRNA was transfected through the lentiviral system. HEK293T cells were transfected with GIPZ lentiviral human *BBS7* shRNA (RHS4430-200209883, Horizon Discovery, United Kingdom) and packaging vectors (pVSVG and psPAX2) using FuGENE at 70% confluency. GIPZ lentiviral shRNA controls (RHS4348, Horizon Discovery, United Kingdom) were used as controls. The viral supernatant was harvested at 48 h post-transfection, filtered through 0.45 μm filters, and applied to human PDL cells. The transfected cells were selected with puromycin (A11138-03, Life Technologies, United States) at 24 h after transfection. Transfected cells were labeled with Turbo GFP.

## Histology

Extracted premolars were immersed in 4% paraformaldehyde (PFA) for cell fixation and then decalcified in 10% EDTA (pH 7.4; BE021, Bio solution Co. Ltd., Korea) for 3 days at 50°C. The paraffin-embedded specimens were sectioned into 7 μm. Hematoxylin and eosin and Masson’s trichrome staining were performed after deparaffinization.

As described in a previous study (Lee et al., 2017), the oxytalan fibers in PDL were detected by the resorcin-fuchsin staining. For the resorcin-fuchsin staining, the slides were oxidized with Oxone^®^ (diluted with distilled water to 10% m/v, 228036, Sigma-Aldrich, Switzerland) for 10 min at room temperature. After rinsing thrice with distilled water (2 min each time), the slides were stained with resorcin-fuchsin solution (26,370, Electron Microscopy Sciences, United States) for 10 min at room temperature. The slides were differentiated in pure ethanol (three changes with 10 immersions each time), rinsed with running water, dehydrated, and mounted. The slides were observed using the stereomicroscope.

For immunofluorescence staining, the slides were incubated with Proteinase K (10 μg/ml, AM2546, Thermo Scientific, United States) for 20 min at 37°C. Subsequently, the slides were incubated with antibodies against acetylated-tubulin (Ac-Tub, 1: 1,000 diluted, T7551, Sigma-Aldrich, United States), vascular endothelial growth factor (VEGF, 1: 200 diluted, sc-53462, Santa Cruz, United States), active RhoA (1: 100 diluted, 26904, NewEast Biosciences, United States), and VEGFR2 (1:100 diluted, 24795, CST, United States) at 4°C overnight. The specimens were sequentially incubated with secondary antibodies (Invitrogen, OR, United States) for 2 h at room temperature and were counterstained with DAPI (D1306, Invitrogen, OR, United States; 30 nM). Fluorescence detection was performed according to the manufacturer’s protocols and examined using a confocal laser microscope (DMi8, Leica, Germany).

## Wound Healing and Tubule Formation Assay

Human PDL cells were seeded at a concentration of 5 × 10^4^ cells per well in a 24-well plate and cultured to achieve complete spreading of cells and 100% monolayer confluency. Gentle cross scratching with a 200-µL tip was applied to each well. GFP-labeled HUVECs were seeded in Matrigel® (50 μL, 354234, Corning, AZ, United States)-coated 96-well plates (2 × 10^4^ cells per well) and cultured with the endothelial cell basal medium (EBM)®-2 (CC-3156, Lonza, Walkersville, MD, United States) supplemented with EGM™-2 SingleQuots® (CC-4176, Lonza, Walkersville, MD, United States) for 24 h. Imaging was performed under 10/×0.25 magnification with CQ-1 inverted fluorescence microscope (CQ-1, Yokogawa, Japan). 195 (migration assay) or 145 serials (tubule formation assay) of two-dimensional confocal images through 517 nm channels were recorded in the environmental chamber, which ensured a constant temperature (37°C), and 5% CO_2_ atmosphere throughout the duration of imaging. All image acquisition settings were identical for the experimental variants in each experiment. The ImageJ plugins “TrackMate” was used to record the trajectory of the PDL cells. The migration distance and wound-healing area were calculated. The cell trajectory plot was analyzed using Chemotaxis and Migration Tool 2.0 (plugin for ImageJ).

## Western Blot

Primary PDL cells were lysed with RIPA buffer supplemented with a proteinase inhibitor cocktail (cOmplete™; #11697498001, Roche, IN, United States). Cell extracts were fractionated by SDS-PAGE and transferred to a polyvinylidene difluoride membrane using a transfer apparatus according to the manufacturer’s protocols (Bio-Rad, California, United States). After blocking with 5% skim milk in TBST (10 mM Tris, pH 7.4, 150 mM NaCl, 0.1% Tween 20) for 60 min, the membrane was incubated with antibodies against Ptch1 (1:1,000 diluted, SC6149, Santa Cruz, United States), Gli1 (1:1,000 diluted, ab49314, Abcam, United Kingdom), active RhoA (1: 1,000 diluted, 26,904, NewEast Biosciences, United States), ROCK 1 (1: 1,000 diluted, ab45171, Abcam, United Kingdom), *α*-smooth muscle actin (*α*-SMA, 1: 100 diluted, 14-9760-80, Invitrogen, United States), von Willebrand factor (vWF, 1: 1,000 diluted, AB7356, Sigma-Aldrich, United States), and GAPDH (1: 1,000 diluted, sc-3233, Santa Cruz, United States) at 4°C overnight. Membranes were washed three times for 15 min and incubated with HRP-conjugated secondary antibodies for 2 h at room temperature. After three washes with TBST, the membranes were developed using the ECL system (RPN2232, GE Healthcare Life Sciences, United States) according to the manufacturer’s protocols.

## Real-Time Quantitative Polymerase Chain Reaction

Total RNA was isolated from human PDL tissues or confluent cell cultures using the TRIzol® reagent. The extract was reverse-transcribed using Maxime RT PreMix (#25081, iNtRON, Korea), and RT-qPCR was performed using the StepOnePlus Real-Time PCR System (Applied Biosystems, United States). The amplification program consisted of 40 cycles of the following: denaturation at 95°C for 50 s, annealing at 60°C for 30 s, and extension at 72°C for 70 s. The RT-qPCR primers for *BBS7*, *PTCH1*, *GLI1*, *COL*, *CD31*, *vWF*, *VEGF*, *PDGF*, and *B2M* (Supplementary Table S1).

## Statistical Analysis

All quantitative results were expressed as the mean ± standard deviation. GraphPad Prism 8 (GraphPad Software, San Diego, CA, United States) was used to analyze all data. Comparisons between two groups were performed using an unpaired two-tailed *t*-test. One-way analysis of variance (ANOVA) with Tukey’s multiple comparison test was performed for multiple group comparisons. Statistical significance was set at *p* < 0.05.

## Results

### Expression of *BBS7* Downregulated in Occlusal Hypofunctional PDL

DEG analysis identified 252 and 318 significantly upregulated and downregulated genes, respectively, in hypofunctional samples (Figure 1B and Supplementary Table S2). GO over-representation analysis (GO ORA) was performed to identify GO terms representing the biological function of the genes (Yu et al., 2012). The analysis identified 71 and 128 significantly enriched GO terms (*p* < 0.05) for the upregulated and downregulated genes, respectively (Supplementary Tables S3, S4). Clustering of the GO terms based on their semantic similarity aids the interpretation of GO analysis by the removal of redundancy (Yu et al., 2010). Clustering suggested totaling 8 and 11 GO clusters for upregulated and downregulated genes, respectively (Supplementary Table S5). Most of the GO clusters contained immune-related GO terms (Supplementary Table S6). Interestingly, clusters consisting of neuron-, osteogenesis-, or extracellular matrix-related terms were found in GO clusters representing significantly downregulated genes in the hypofunction group (Figure 1C). These results are consistent with the known effects of occlusal hypofunction, such as the degeneration of neurons, alveolar bone loss, and periodontal ligament damage (Kaneko et al., 2001; Shimomoto et al., 2007; Aung et al., 2020). To discover novel genes related to tissue homeostasis and regeneration, we investigated the top-ranked genes in the significantly downregulated gene group (Figure 1D). Among the top 20 downregulated genes, we found *BBS7*, a gene encoding one of seven proteins of the BBSome complex. BBSome is involved in primary cilia function and is essential for the Shh signaling transduction (Zhang et al., 2012), which were related to tissue regeneration (Irigoin and Badano, 2011). Moreover, the symptom of Bardet-Biedl syndrome included oral structure and dental anomalies such as crowding, hypodontia, small roots, and high-arched palate (Panny et al., 2017). We examined the expressions of related genes encoding BBS proteins; however, only *BBS7* was significantly regulated by the absent of the occlusal force (Figure 1E). Based on the RNA-seq result, we set about investigating the relationship between *BBS7* and tissue homeostasis.

## Morphology Alternation in Occlusal Hypofunctional PDL

**FIGURE 2:**
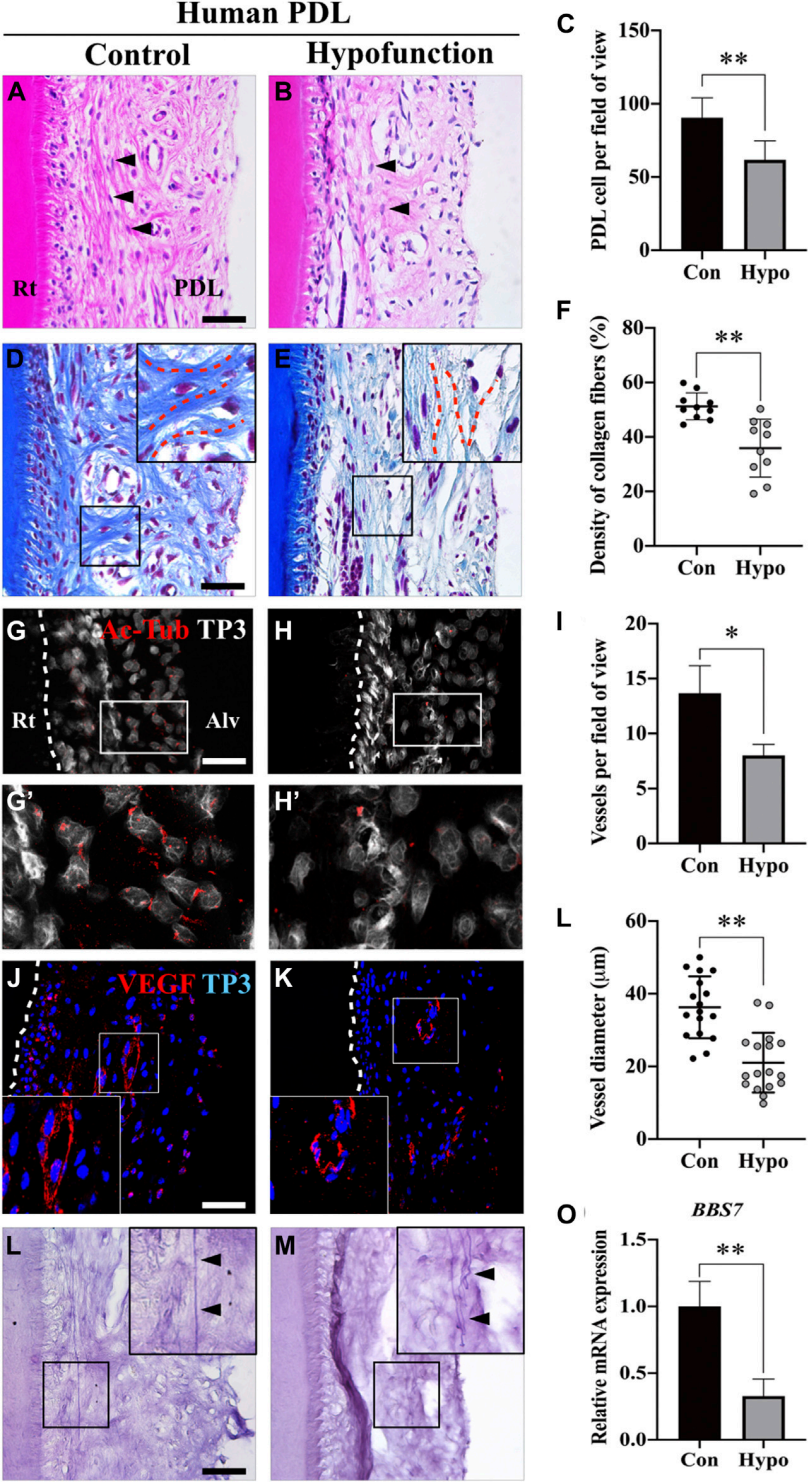
Morphological changes of PDL in patients with occlusal hypofunction. **(A,B)** Hematoxylin and eosin staining of human PDL from the extracted tooth. The shape of fibroblasts (arrowheads) was changed from spindle (control) to round (hypofunction). **(C)** Quantification of PDL cell number in control and hypofunction groups (*n* = 5). **(D,E)** Masson’s trichrome staining showed disorientation of the type I collagen fibers (blue) in the hypofunction group. The PDL fibers aligned perpendicular to the root in the control group, whereas the relatively sparse fibers were parallel to the root in the hypofunction group (red dotted line). **(F)** Quantification of type I collagen fiber density in control and hypofunction groups. **(G,H)** Primary cilia were stained with Ac-Tub, and nuclei were stained with TP3. White dotted line indicated the margin of the root. (G′) Ac-Tub–positive cells in the control group were gathered along the alveolar bone side (H′), whereas it was rarely observed in the hypofunction group. **(I,J)** Blood vessels in PDL were stained with VEGF, and the nuclei were stained with TP3. White box magnified the well-organized (control) or disrupted (hypofunction) blood vessel. **(K)** The number of blood vessels decreased by one-third in the hypofunction group compared to the control group (*n* = 3). **(L)** The diameter of blood vessels in the hypofunction group was reduced by 40% compared to the control group (*n* = 30). **(M,N)** Oxytalan fibers were detected by the resorcin-fuchsin staining. The arrowheads indicated straight and extensive fibers in the control group, and tortuous fibers in the hypofunction group. **(O)** Relative mRNA expression of *BBS7* in PDL tissue (N = 3); scale bars: A, B, D, E, G, H, M, N, 100 μm; I, J, 50 μm. Quantitative data were presented in mean ± SD. *p < 0.01, **p < 0.001. Rt: root; PDL: periodontal ligament; Alv: alveolar bone; Con: control group; Hypo: hypofunction group; Ac-Tub: acetylated-tubulin; TP3: TO-PRO-3; VEGF: vascular endothelial growth factor.

To investigate the morphological differences between the control and occlusal hypofunctional PDL, the apical third tooth was histologically evaluated. Spindle-shaped fibroblasts were observed in the control group, whereas they appeared round in shape in the hypofunction group (Figures 2A,B). The number of PDL cells was quantified, which showed a one-third decrease in the hypofunction group versus in the control group (Figure 2C). Masson’s trichrome staining in the hypofunction group revealed the disorientation of collagen fibers, which were aligned parallel to the root, while the fibers in the control group were perpendicular to the root. The interstitial space, including blood vessels, was rarely observed in the hypofunction group compared to the control group (Figures 2D,E). The density of type I collagen fibers decreased by approximately 30% in the hypofunction group compared to the control group (Figure 2F). The distribution of primary cilia in PDL tissue was examined using acetylated-tubulin (Ac-Tub) (Figures 2G,H). The primary cilia protruded from the PDL cell surface, which gathered along the alveolar bone in the control group. Primary cilia were rarely observed in the hypofunction group (Figure 2G’, H′). The blood vessels in the interstitial space of the PDL were examined using vascular endothelial growth factor (VEGF) (Figures 2I,J). Relatively well-organized blood vessels were observed in the control group compared to those in the hypofunction group. Quantification analysis showed that the mean number of blood vessels and the diameter in the hypofunction group were decreased when compared with the control group, by 41.6 and 42.9%, respectively (Figures 2K,L). The oxytalan fibers in the control group were straight and extensive (Figure 2M). In contrast, the tortuous fibers were observed in the hypofunction group (Figure 2N). To confirm the *BBS7* mRNA expression in human PDL tissue, RT-qPCR was performed. *BBS7* significantly reduced in the hypofunctional PDL (Figure 2O). Together, these results reveal that occlusal hypofunction leads to a dynamic alternation in PDL, including the cells, fibers, primary cilia, and blood vessels.

## *BBS7* Knockdown Delays the Cell Migration

**FIGURE 3:**
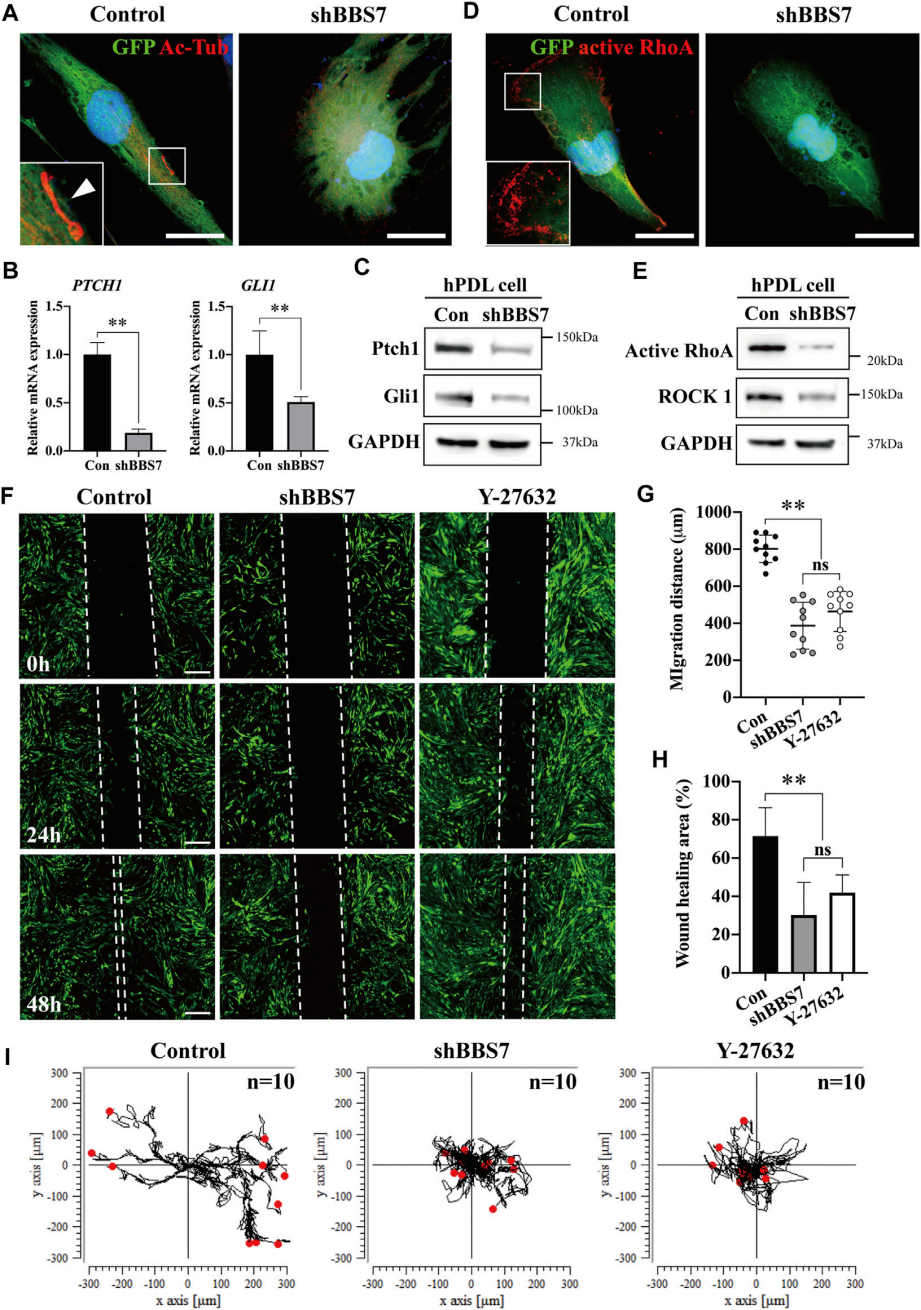
Effect of *BBS7* knockdown on the primary PDL cell migration. **(A)** GFP-labeled PDL cells are stained with Ac-Tub (red), and nuclei are stained with TO-PRO-3 (blue). The primary cilia (arrowhead) were observed in the control group. PDL cells were spindle-shaped in the control group, whereas they were round in shape in the shBBS7 group. **(B)** Relative mRNA expression of Shh signaling molecules. Compared to the control group, the expression of *PTCH1* and *GLI1* was significantly decreased in the shBBS7 group. **(C)** Immunoblotting with Ptch1, Gli1, and GAPDH antibodies. The Ptch1 and Gli1 protein expressions were significantly decreased in the shBBS7 group. **(D)** Actomyosin cytoskeleton–related kinase is stained with active RhoA (red), and nuclei are stained with TO-PTO-3 (blue). **(E)** Immunoblotting with active RhoA, ROCK1, and GAPDH antibodies. The active RhoA and ROCK1 protein expressions were significantly decreased in the shBBS7 group. **(F)** PDL cell migration was examined by wound-healing assay (control, shBBS7, and Y-27632 group). The scratched and recovering of wounded areas were marked by white dotted lines. Representative images were captured at 0, 24, and 48 h. The shBBS7 group showed delayed wound closure compared to control and Y-27632 groups. **(G)** Quantification of migration distance in shBBS7 and Y-27632 groups was significantly lower than the control group. **(H)** The wound-healing area of shBBS7 and Y-27632 groups was significantly lower than the control group. **(I)** The representative trajectory plots depicting the path of the individual PDL cell movement. The trajectories were displayed by using position parameters and shifted to a common origin. The directional movement was observed in the control group, while the cell in shBBS7 and Y-27632 groups had low persistence to migration; scale bars: A, D, 25 μm; F, 400 μm **p < 0.001. Ac-Tub: acetylated-tubulin; Con: control.

To investigate the relationship between *BBS7* and Shh signaling activity, we utilized lentiviral transduction for knockdown of *BBS7* in primary PDL cells (shBBS7 group). Primary cilia were only observed in spindle-shaped PDL cells of the control group, whereas the cells were round in shape in the shBBS7 group (Figure 3A). *BBS7* knockdown was confirmed through RT-qPCR (Supplementary Figure S1A). *COL1* also significantly decreased in the shBBS7 group (Supplementary Figure S1B), which was consistent with the RNA-seq results and belongs to cluster five in GO analysis (Figure 1C, Supplementary Table S3 Go:0030199). It has been reported that the non-canonical Hedgehog signaling pathway regulates the cell migration through the activation of RhoA (Robbins et al., 2012). Relative mRNA and protein expression of *PTCH1* and *GLI1* were dramatically decreased in the shBBS7 group (Figures 3B,C). The result indicated that Shh signaling activity was inhibited by *BBS7* knockdown. The expression of active RhoA in the shBBS7 group significantly decreased compared to the control group (Figure 3D). Compared with the control group, protein expressions of active RhoA and ROCK1 were significantly decreased in the shBBS7 group (Figure 3E). To confirm the effect of *BBS7* on cell migration, the wound-healing assay was performed. Additionally, the Y-27632 (ROCK1 inhibitor) treatment group was used to compare the cell migration capability. The wounding scratches were captured at different time points (0, 24, 48 h, and 15 min intervals) (Figure 3F, Supplementary Videos S1, S2, S3). Wound enclosure was observed in a time-dependent manner in all the groups. The shBBS7 group showed dramatically delayed cell migration at 24 and 48 h compared with the control and Y-27632 groups. The migration distance revealed 50% decrease in the shBBS7 group and 37.5% decrease in the Y-27632 group when compared to the control group (Figure 3G). The wound-healing area in the hypofunction and Y-27632 groups was also significantly less than that in the control group (57 and 44.5%, respectively) (Figure 3H). The individual cell movement was transformed in the trajectory plots (Figure 3I). Directional migration was observed in the control group, whereas the cells in the shBBS7 and Y-27632 groups showed low persistence of directional migration. According to the above results, BBS7 plays a crucial role in the directional migration of PDL cells.

## *BBS7* Knockdown Suppresses the Cell Angiogenesis in PDL

**FIGURE 4:**
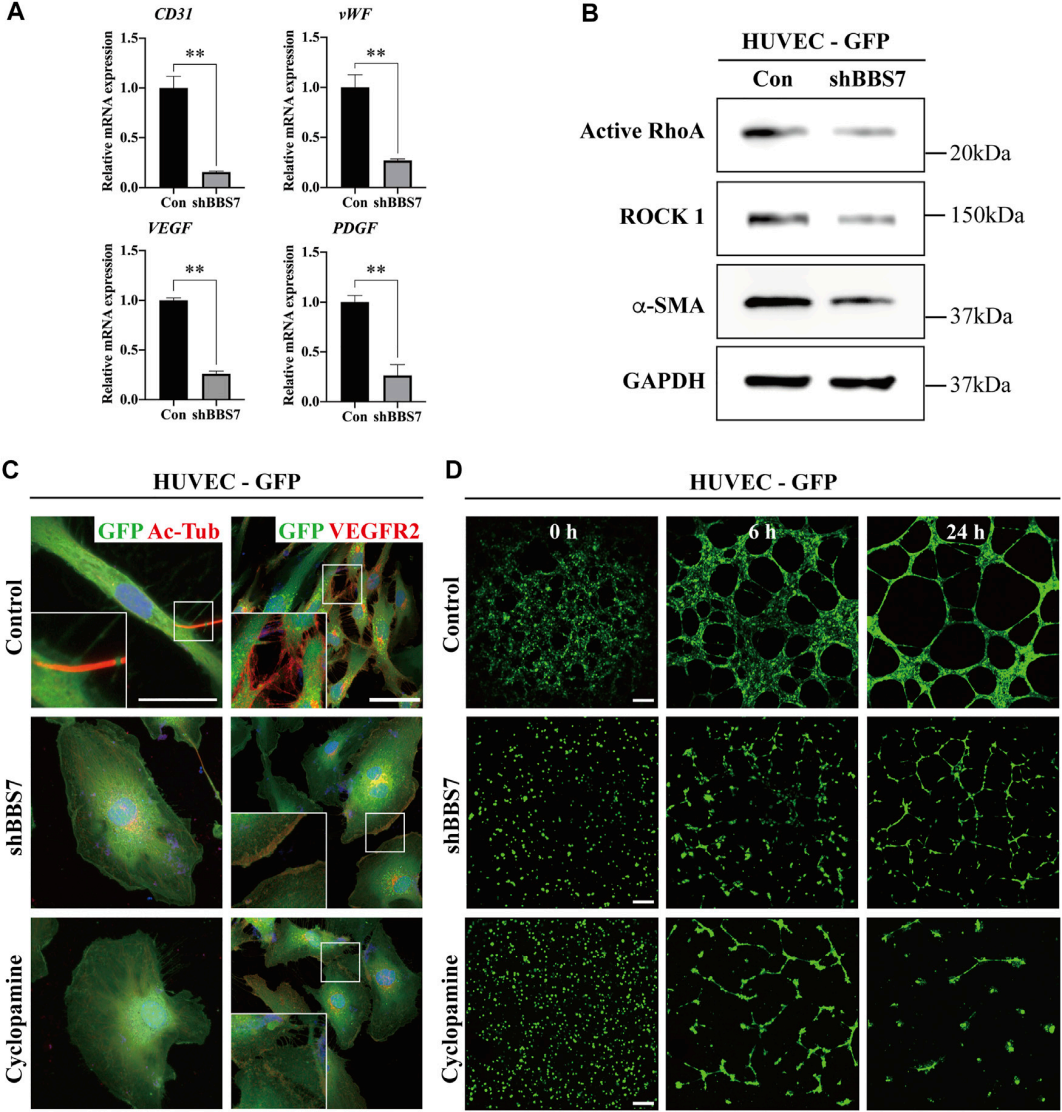
Effect of *BBS7* knockdown on HUVECs. **(A)** Relative mRNA expression on angiogenesis markers. Compared to the control group, the expression of *CD31*, *vWF*, *VEGF*, and *PDGF* is significantly decreased in the shBBS7 group. **(B)** The protein expression of migration markers (active RhoA and ROCK 1) and endothelial marker (*α*-SMA) is significantly decreased in the shBBS7 group. **(C)** Ac-Tub–labeled primary cilia (red) are only observed in the control group. The expression of VEGFR2 is significantly decreased in shBBS7 and cyclopamine groups compared to the control group. **(D)** GFP-labeled HUVECs are cultured for 24 h in a 96-well plate coated with Matrigel^®^. Cells of the control group initially attached at 0 h, then migrated individually over the next 6 h and formed tubule-like structures, which matured by 24 h. Cells of the shBBS7 group initially attached at 0 h, then just a few tubule-like structures at 6 h, and failed tubule formation at 24 h. Cyclopamine group failed to form a junction and network at 6 and 24 h. Con: control group; scale bars; C. left column, 25 μm; right column, 50 μm. D, 400 μm, Ac-Tub: acetylated-tubulin; VEGFR2: vascular endothelial growth factor receptor 2.

In general, the blood vessels are abundantly distributed in the PDL interstitial space. A previous study reported that the number of vessels in PDL is seven to ten times greater than that in other fibrous connective tissues (Sims, 1987). Interestingly, we observed that the number of blood vessels as well as the diameter decreased in the occlusal hypofunctional PDL. We utilized shRNA to further investigate whether the *BBS7* knockdown could affect angiogenesis. BBS7 knockdown was performed in HUVECs (shBBS7 group) to evaluate the mRNA and protein expression. The relative mRNA expression of endothelial marker (*CD31*) and angiogenesis markers (*vWF*, *VEGF*, and *PDGF*) was significantly decreased in the shBBS group (Figure 4A). In addition, cell migration plays a pivotal role in angiogenesis (Lamalice et al., 2007). We confirmed that the migration markers (active RhoA and ROCK 1) and endothelial markers (*α*-SMA) were downregulated in the shBBS7 group (Figure 4B). The tubule formation assay was performed using GFP-labeled HUVECs. Additionally, the cyclopamine (Shh pathway antagonist, 10 μg/ml) treatment group was used to compare the tubule formation ability. Primary cilia and VEGFR2 were presented in the control group but not in the shBBS7 and cyclopamine groups (Figure 4C). Time-lapse images of tubule formation in HUVECs with control, shBBS7, and cyclopamine groups were taken for 24 h (Supplementary Videos S4, S5, S6). Thirty minutes after seeding HUVECs on Matrigel^®^-coated 96-well plate, enough time was allowed for the cells to sink and attach to the plate, and this was set to 0 h. At 0 h, HUVECs with control infection formed a meshwork structure (Figure 4D). However, shBBS7 and cyclopamine groups showed an evenly distributed attachment. The cells were aligned and formed tubule-like structures at 6 h, and the network matured at 24 h with the appearance of a lumen structure in the control group. In contrast, the shBBS7 group showed a disconnected network at 6 h, and the tubule forming totally failed at 24 h. The cyclopamine group also fails to form a junction and network structure during 24 h observation. The junctions and tubule formation were quantified using Carpentier’s Angiogenesis Analyzer for the ImageJ plugin. The numbers of matured junctions and tubules were not observed in the shBBS7 and cyclopamine groups (Supplementary Figure S2B, C). Based on the above result, the absence of *BBS7* suppresses angiogenesis through interrupted cell migration.

## Discussion

The present study investigated the relationship among occlusal force, *BBS7*, primary cilia, and Shh signaling activity in human PDL tissue. Based on the transcriptome analysis, the expression of *BBS7* was found to be significantly downregulated in occlusal hypofunctional PDL. In addition, morphological changes, including fibroblasts, type I collagen fibers, and blood vessels, were observed in occlusal hypofunctional PDL. These observations are analogous to those of previous studies using mouse models (Usumi-Fujita et al., 2013; Itohiya et al., 2016; Kasahara et al., 2017). These indicate that *BBS7* may be strongly involved in the atrophic changes in PDL which is induced by the absence of mechanical stimulation. The oxytalan fiber is an elastic fiber within the PDL that emerges from the cementum, generally runs in an occluso-apical direction, and attaches to the blood vessels. It is responsible for the maintenance and support of the vascular system (Strydom et al., 2012). In the present study, tortuous oxytalan fibers were observed in occlusal hypofunctional PDL. Hence, the atrophic changes in blood vessels were strongly associated with the deformed oxytalan fibers that were speculated.

BBS7 is a constituent of a protein complex named the BBSome, which participates in the sorting and trafficking of membrane proteins into primary cilia (Jin et al., 2010). BBS7 is indispensable in the BBSome assembly (Seo et al., 2011). The mutation of bbs7 and bbs8 in *Caenorhabditis elegans* results in shortened cilia and abnormity of ciliary cargo trafficking (Blacque et al., 2004). As a signaling sensory organelle, primary cilia play a central role in the transduction of Shh signals. Abnormities in the biogenesis of transport through primary cilia influence Shh signaling, and the Ptch1 and Smo accumulate in cilia (Zhang et al., 2012). To investigate the relationship among *BBS7*, primary cilia, and Shh signaling in PDL homeostasis, we applied the shRNA system to knock down the *BBS7* gene in human primary PDL cells.

The relative mRNA expression of Shh signaling components in the *BBS7* knockdown PDL cells was examined, and the decreased mRNA and protein expression levels of *PTCH1* and *GLI1* indicated that *BBS7* regulates Shh signaling activity. Shh receptor Ptch1 negatively regulates the activity and ciliary accumulation of Smo, which is essential for transducing Shh signal (Kim et al., 2015). The knockdown of *BBS7* in PDL cells may affect the exit of Ptch1 from the cilia, which eventually results in the downregulation of Shh signaling activity.

Cell migration is a highly coordinated multistep process that plays a crucial role in tissue homeostasis and regeneration (Guo and Rahmouni, 2019). In addition, a number of studies have reported that the non-canonical Shh signaling pathway mediates cell migration through the activation of small GTPases, such as RhoA (Robbins et al., 2012). PDL cell migration was significantly inhibited by *BBS7* knockdown. The protein expression of active RhoA and its downstream effector ROCK1 also decreased. Furthermore, after *BBS7* knockdown, the cell morphology changes from a spindle to a round shape. These results indicate that the *BBS7* may regulate the PDL cell migration through non-canonical Shh signaling. Cell migration is a critical step during angiogenesis (Ouchani et al., 2015). Angiogenesis includes destabilization of the integrated blood vessel, proliferation, migration, invasion, and tubulogenesis of endothelial cells. It is well known that in PDL tissue there are various resident cell population including fibroblast, cementoblast, osteoblast, osteoclast, endothelial cell, and undifferentiated mesenchymal cells. We found out that the vessel structure was disrupted in hypofunctional PDL (Figures 2I,J). Moreover, it was confirmed that cell migration was suppressed by the downregulation of BBS7, as shown in Figure 3. Cell migration is essential for the sprouting angiogenesis in tissue homeostasis and regeneration (Lamalice et al., 2007). Hence, we hypothesized that the loss of angiogenesis may also be related to the downregulation of *BBS7*. For supporting the hypothesis, we not only additionally analyzed the mRNA expression of angiogenesis-related marker (PDGF, VEGF, and vWF) and the endothelial cell marker CD31 but also tested the protein expression of migration-related marker (Active RhoA, ROCK 1) and endothelial marker (a-SMA) in HUVECs, which is one of the most frequently used cell lines in angiogenesis research (Figures 4A,B). All marker expressions were significantly decreased. VEGF and VEGFR2 have been recognized as important angiogenic molecules. The previous study showed that Shh simulation promoted the phosphorylation of VEGF2 during angiogenesis (Lei et al., 2020). Cyclopamine-antagonizing Smo inhibits Shh signaling activity (Taipale et al., 2000). We used shRNA to knockdown *BBS7* in HUVECs. The tubule-forming ability and VEGFR2 expression were dramatically decreased in *BBS7* knockdown in HUVECs. In addition, after direct inhibition of the Shh signaling pathway by treatment with cyclopamine, HUVECs’ tubule formation was dramatically suppressed, which is analogous to the results of the *BBS7* knockdown group. To sum up, the reduction of BBS7-SHH signaling axis inhibits the endothelial cell migration which can eventually affect the angiogenesis in PDL.

## Conclusion

**FIGURE 5:**
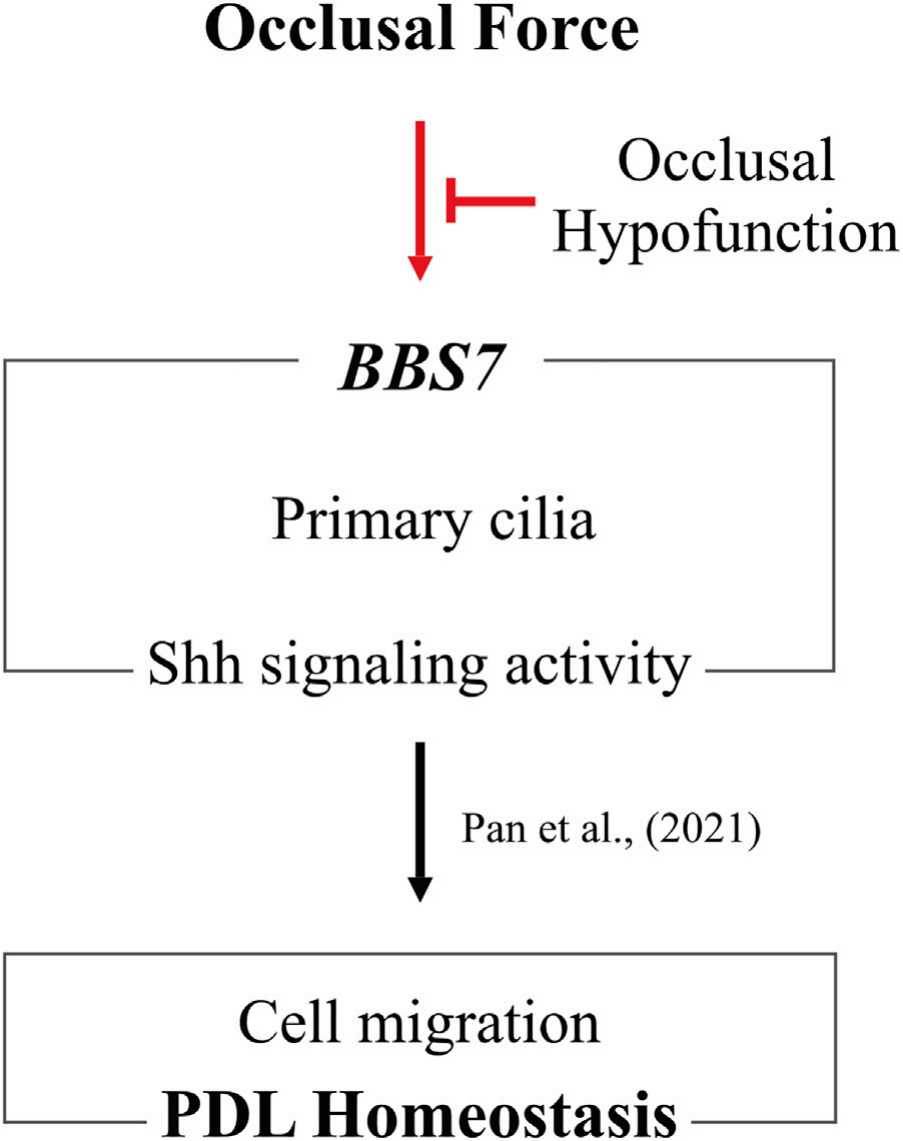
Occlusal hypofunction affects PDL homeostasis through downregulating *BBS7*. Maintenance of *BBS7* expression with Shh signaling activity in primary cilia is of fundamental importance by occlusal force. Occlusal hypofunction is closely associated with these relationships. Furthermore, to sustain PDL homeostasis, proper occlusal force is indispensable.

In conclusion, *BBS7* plays an essential role in PDL homeostasis (Figure 5). Occlusal force regulates the expression of *BBS7* to mediate Shh signaling activity, which orchestrates cell migration for proper PDL homeostasis. However, the present study could not elucidate how each *BBS* family gene is closely associated with occlusal force. The current human study provides fundamental importance to explain how vigorous PDL homeostasis is maintained by occlusal force.

## Data Availability

The data presented in the study are deposited in the GEO repository, accession number GSE189288 https://www.ncbi.nlm.nih.gov/geo/query/acc.cgi?acc=GSE189288
