# Supplementary material for: BBS7–SHH Signaling Activity Regulates Primary Cilia for Periodontal Homeostasis
Source: Front Cell Dev Biol. 2021 Dec 7;9:796274. doi: 10.3389/fcell.2021.796274 (PMC8703258; doi:10.3389/fcell.2021.796274)
Supplement: Supplementary file 4 [file Presentation1.pdf]

BBS7 - SHH signaling activity regulates primary cilia for periodontal homeostasis

Running title: Role of *BBS7* in periodontal homeostasis

Pi En Chang, Shujin Li, Hyun-Yi Kim, Dong-Joon Lee, Yoon Jeong Choi, Han-Sung Jung

Supplementary file contents:

Appendix materials and methods

Figure S1. Relative mRNA expression of *BBS7 and COL1* in *BBS7* knockdown primary PDL cells.

Figure S2. Relative mRNA expression of *BBS7*, number of junctions and tubes in *BBS7* knockdown HUVECs.

Table S1. RT-qPCR primer sequence.

Table S2. List of significant differentially expressed genes (DEGs) in occlusal hypofunction samples compared with control samples.

Table S3. List of significantly enriched gene ontology (GO) terms of significantly up-regulated DEGs in occlusal hypofunction samples compared with control samples.

Table S4. List of significantly enriched GO terms of significantly down-regulated DEGs in occlusal hypofunction samples compared with control samples.

Table S5. List of GO clusters of significantly up-regulated DEGs in occlusal hypofunction samples compared with control samples.

Table S6. List of GO clusters of significantly down-regulated DEGs in occlusal hypofunction samples compared with control samples.

Video S1. Human PDL cell wound healing assay (control group).

Video S2. Human PDL cell wound healing assay (shBBS7 group).

Video S3. Human PDL cell wound healing assay (Y-27632 group).

Video S4. HUVECs tubule formation assay (control group).

Video S5. HUVECs tubule formation assay (shBBS7 group).

Video S6. HUVECs tubule formation assay (Cyclopamine group).

Supplementary Figures

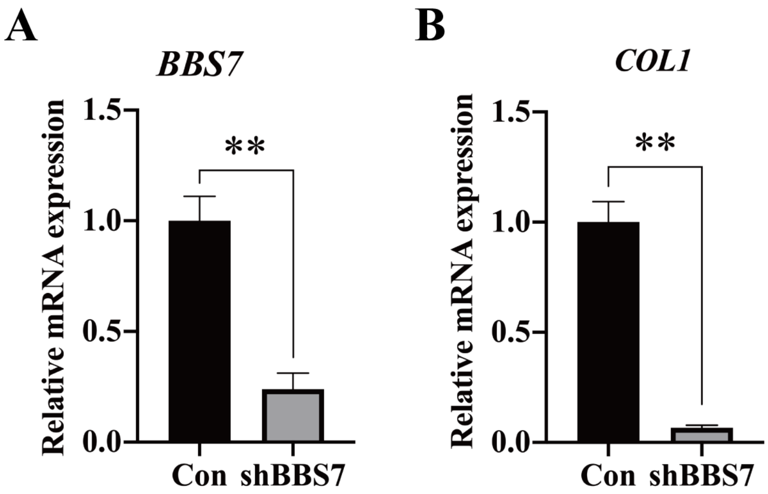

Figure S1 Relative mRNA expression of *BBS7, COL1* in *BBS7* knockdown primary PDL cells.

n=3 (A, B) Compare to the control group, *BBS7* and *COL1* mRNA expression significantly decreased in the hypofunction group. ***p* value <0.001. Con: control group, Hypo: hypofunction group.

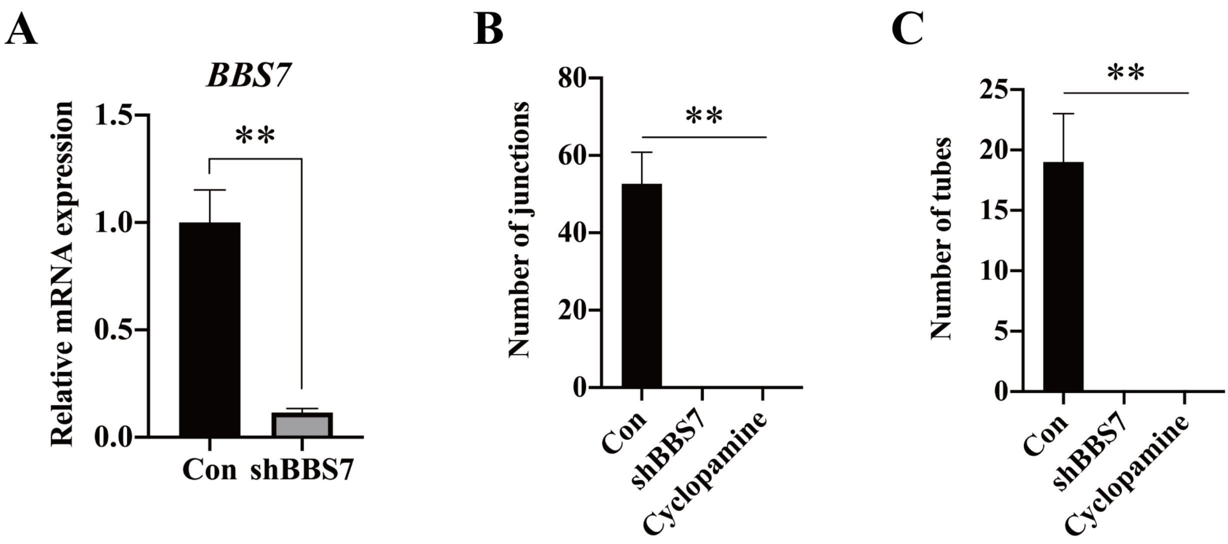

Figure S2. Relative mRNA expression of *BBS7*, number of junctions and tubes in *BBS7* knockdown HUVECs. (A) The relative mRNA expression of *BBS7* was significantly decreased in the shBBS7 group. (B, C) The numbers of junctions and tubules were analyzed by ImageJ plugin: Carpentier's Angiogenesis Analyzer. Junction and tubule in shBBS7 and Cyclopamine groups were not counted.

*p value<0.01, **p value<0.001

Supplementary Tables

Table S1 RT-qPCR primer

BBS7	F5´-ATCAACAGTAGTTCAAGGTGCC-3´	R5´-CACGGAGCTCACATAGTAATACAG-3´
SHH	F5´-TTATCCCCAATGTGGCCGAG-3´	R5´-TTACACCTCTGAGTCATCAGCC-3´
PTCH1	F5´-GGAGCAGATTTCCAAGGGGA-3´	R5´-CCACAACCAAGAACTTGCCG-3´
SMO	F5´-TGAAGGCTGCACGAATGAGG-3´	R5´-CTTGGGGTTGTCTGTCCGAA-3´
GLI1	F5´-CCCATTCCAATGAGAAGCCG-3´	R5´-AGAAATGGATGGTGCCCGAG-3´
COL1	F5´-CTGTAAACTCCCTCCATCCC-3´	R5´-GTCCATGTGAAATTGTCTCCC-3´
CD31	F5´- GCTGAGTCTCACAAAGATCTAGGA-3´	R5´- ATCTGCTTTCCACGGCATCA-3´
vWF	F5´- GCTGCTGGACACAAGTTTGA-3´	R5´- ACTCATGGGGCTCTGCATAC-3´
VEGF	F5G- AGATCGAGTACATCTTCAAGCCATC-3G	R5´- CGTCATTGCAGCAGCCC-3´
PDGF	F5´- GAAGGCCAGGGAGTCAGGT-3´	R5´- TCACATCTGGGCTGCTT3´
B2M	F5´-TGTCTGGGTTTCATCCATCCG-3´	R5´-TTCACACGGCAGGCATACTC-3´

Table S2. List of significant differentially expressed genes (DEGs) in occlusal hypofunction samples compared with control samples.

	Symbol	Log2FC	pVal		Symbol	Log2FC	pVal		Symbol	Log2FC	pVal
1	FAM217A	9.262344	3.46E-05	29	S100A7	1.858332	1.41E-143	57	IGHV5-51	1.205387	3.21E-127
2	BAALC	6.754235	1.56E-07	30	SEMA4D	1.840805	2.55E-41	58	IGHV2-5	1.20516	5.79E-130
3	AL669831.4	5.877777	2.91E-16	31	IGKV1-39	1.738802	9.50E-05	59	AC244230.1	1.203482	3.26E-10
4	DNAI1	4.964252	6.30E-05	32	NIPAL2	1.721287	1.19E-29	60	AC087276.1	1.203369	1.16E-07
5	SYNGAP1	4.817444	3.21E-207	33	LPIN1	1.618511	2.59E-32	61	IL36A	1.196023	4.25E-07
6	ADAMTS7P3	3.975308	0.001209	34	IGHV3-11	1.61129	2.40E-137	62	OCIAD2	1.186118	9.71E-14
7	IGKV1D-42	3.738397	0.00482	35	IGHV3-66	1.595596	1.42E-46	63	PRDM5	1.176153	4.19E-25
8	IGHV1-69	3.634243	8.02E-55	36	IGLV6-57	1.595434	8.69E-94	64	CXCL13	1.173331	6.34E-49
9	SP5	3.315068	0.037682	37	SNAP23	1.591692	4.29E-33	65	IGLV4-69	1.166597	7.51E-29
10	AC107918.4	3.280212	0.012921	38	IGLV1-36	1.582748	4.62E-78	66	IGKV1-6	1.139424	2.98E-98
11	IMMP1L	2.918567	1.97E-256	39	ARL14EP	1.555892	5.03E-38	67	IGLV2-18	1.125627	2.50E-29
12	EFL1	2.803753	2.08E-154	40	IGHV4-61	1.523325	1.04E-110	68	AC005943.2	1.125505	4.26E-190
13	CKMT1B	2.793467	7.44E-09	41	IL6	1.5231	0.017398	69	IGKV1-8	1.122501	0.015211
14	AC244669.1	2.736437	1.52E-32	42	IGHV3-53	1.503483	3.56E-30	70	IGHV3-49	1.066313	5.65E-26
15	MTFMT	2.715437	5.80E-86	43	LCE3D	1.472751	0.001204	71	IGHG4	1.038188	2.00E-204
16	IGHV3-20	2.609961	5.61E-47	44	CCL18	1.462445	3.94E-05	72	APOC1	1.034137	2.12E-05
17	LBP	2.549291	5.22E-07	45	CLEC4E	1.450238	0.002991	73	IMMP2L	1.027674	5.51E-08
18	IGLV3-10	2.347916	1.02E-196	46	AC068213.1	1.446325	0.00012	74	IGKV1-17	1.017283	3.49E-64
19	IGKV1-12	2.296109	0	47	IGLV4-60	1.436669	1.75E-07	75	IGHV3-21	1.014306	4.12E-89
20	KHDRBS3	2.276738	1.95E-36	48	IGKV1-9	1.358624	1.92E-152	76	FAM25C	1.014305	7.87E-08
21	ZNF385B	2.207591	8.69E-08	49	IGKV3D-11	1.35502	0.017657	77	KDM6B	1.006278	4.28E-11
22	CD177	2.157752	3.19E-11	50	MLLT10	1.319259	8.15E-27	78	SPRR1A	1.005356	2.00E-204
23	CDKL1	2.121933	5.53E-23	51	IGHV1-69D	1.308207	2.98E-166	79	IGKV4-1	1.003013	4.59E-157
24	IGHJ4	2.113465	0	52	DUSP16	1.293615	4.44E-09	80	RAPGEF6	1.000802	3.90E-28
25	AC112484.4	2.065469	4.96E-11	53	G3BP1	1.266551	1.65E-47	81	POU2AF1	0.983954	0.003377
26	ZNF573	2.059893	4.60E-18	54	PLAC8	1.264234	0.005258	82	LCN2	0.979277	2.22E-11
27	IGFLR1	1.991921	2.60E-19	55	IGLV1-44	1.256389	5.83E-168	83	CYP24A1	0.970636	0.007112
28	IGHV2-70	1.876707	1.18E-131	56	ANG	1.221741	0.000138	84	CHI3L1	0.953379	0.031942

	Symbol	Log2FC	pVal		Symbol	Log2FC	pVal		Symbol	Log2FC	pVal
85	CLCA4	0.935812	0.042056	118	GRAMD4	0.72572	0.004643	151	IGLL5	0.578031	9.30E-25
86	PIK3CA	0.930976	5.62E-06	119	IGKV1-27	0.715302	1.35E-29	152	CCL20	0.566309	0.007849
87	IGHV1-24	0.922936	1.44E-69	120	CD79A	0.708497	0.010272	153	IGLV3-27	0.565053	0.044425
88	RNF207	0.912212	1.32E-05	121	ADIRF	0.708002	1.08E-05	154	MMP12	0.56168	7.07E-11
89	IGKV2-30	0.906228	1.10E-19	122	IGHV4-39	0.700406	2.79E-41	155	IGKV2D-29	0.560957	0.007343
90	MZB1	0.904379	9.12E-08	123	IGKV3D-20	0.698322	2.56E-05	156	PI3	0.55948	8.45E-10
91	SPRR2F	0.894416	2.58E-24	124	DERL3	0.697075	1.04E-05	157	IGHG2	0.550183	3.82E-184
92	IGLV2-23	0.860088	1.37E-55	125	IGLV8-61	0.692837	5.12E-21	158	SAMD4A	0.548662	0.020226
93	IGLC3	0.853262	5.91E-98	126	TMPRSS4	0.688602	0.006321	159	IGKV3-20	0.546195	1.19E-107
94	UNKL	0.851919	2.68E-09	127	LINC00674	0.678569	2.26E-27	160	CPM	0.542133	0.013399
95	PRSS22	0.848969	4.75E-12	128	NFATC2	0.676637	0.000153	161	IGHV3-74	0.523949	1.53E-09
96	CCDC137	0.841096	0.008343	129	PDZK1IP1	0.674925	1.12E-06	162	CNFN	0.516946	1.60E-07
97	PIM2	0.829052	0.0005	130	MARK1	0.671622	0.001715	163	ECM1	0.511417	6.99E-16
98	IGHG1	0.823648	0	131	IGHV3-43	0.667803	5.56E-05	164	ANKRD18B	0.51089	0.041081
99	CLDN4	0.820636	3.94E-06	132	SPRR2G	0.666867	0.00789	165	TACSTD2	0.510373	7.76E-21
100	IGKC	0.814034	0	133	IGLC2	0.666253	1.60E-290	166	COX6B2	0.509307	0.000167
101	IGKV1-5	0.812307	6.34E-120	134	MDGA1	0.66356	2.20E-09	167	IGHV3-73	0.507777	8.19E-05
102	IGKV1-16	0.801934	2.21E-41	135	IGLV2-14	0.657839	6.74E-58	168	ERO1A	0.506587	0.000283
103	ARMH4	0.797832	0.000389	136	IGHV4-59	0.654449	1.14E-32	169	FDCSP	0.504917	0
104	UBE2S	0.795747	0.002146	137	IGHV3-7	0.649506	2.50E-71	170	CXCR4	0.500553	0.016431
105	SSBP4	0.79013	7.46E-12	138	IGLV10-54	0.649373	0.031942	171	SPIDR	0.495313	1.39E-13
106	IGHV1-18	0.789384	4.77E-69	139	RHCG	0.644992	2.49E-28	172	TMPRSS11E	0.49345	0.00275
107	SAA1	0.787853	2.88E-05	140	IGHV3-23	0.639989	3.08E-89	173	FXYD3	0.481336	2.44E-06
108	IGLV1-40	0.7872	9.97E-100	141	HDAC11	0.634727	0.023607	174	SLPI	0.474569	4.77E-13
109	TDRP	0.778251	8.36E-08	142	HIVEP2	0.631024	0.028195	175	IGLV7-46	0.462452	8.86E-07
110	DUOX2	0.770838	0.017643	143	IGHV1-58	0.625848	0.043144	176	IL36G	0.461219	0.0001
111	STRA6	0.749389	0.001449	144	IGHV6-1	0.625496	1.46E-06	177	CLDN7	0.461139	0.033663
112	IGLV1-51	0.749259	1.59E-36	145	IGLV5-45	0.622047	3.29E-14	178	COX7A1	0.458966	0.000149
113	IGLV3-21	0.748469	7.04E-24	146	PRSS8	0.603819	0.00578	179	LY6D	0.455916	0.000598
114	DNASE1L3	0.739292	6.26E-24	147	OSGIN1	0.603057	2.78E-05	180	TNN	0.455342	2.64E-27
115	IGKV3-11	0.737703	3.85E-110	148	IGHV4-34	0.598803	2.17E-09	181	ABCA2	0.454266	0.00105
116	SLAMF7	0.736414	0.040952	149	TGM1	0.594842	8.44E-07	182	IGLC1	0.453348	3.03E-14
117	IGHV3-48	0.731643	2.26E-42	150	IGKV3-15	0.580507	2.20E-19	183	CDC42SE2	0.446972	0.033863
184	IGLV1-47	0.437606	8.55E-15	239	HLA-B	0.211609	3.49E-16	272	C1S	-0.12963	0.003583
185	IGLV2-11	0.432464	2.80E-20	240	DKK3	0.190997	0.00013	273	RPS27A	-0.13011	3.11E-06

	Symbol	Log2FC	pVal		Symbol	Log2FC	pVal		Symbol	Log2FC	pVal
186	SOD2	0.42507	2.12E-16	241	NUMA1	0.184817	0.015105	274	H3F3A	-0.13019	0.025127
187	HLA-DQA1	0.421409	0.00082595	242	EMP1	0.167977	0.000409	275	RPLP1	-0.13046	0.000317
188	ST6GALNAC2	0.418207	0.00996852	243	AQP1	0.163229	0.006485	276	RPS15A	-0.13209	5.00E-11
189	S100A8	0.410249	2.44E-141	244	LAMB3	0.147504	0.011682	277	RPL36	-0.13226	0.04258
190	PGM3	0.409222	0.0006849	245	FTH1	0.146048	1.23E-07	278	RPL3	-0.13284	3.39E-08
191	PLXNA3	0.405312	1.09E-09	246	CD9	0.144998	4.81E-05	279	RPL4	-0.13294	1.76E-13
192	XBP1	0.401849	5.51E-09	247	SAT1	0.140434	0.00157	280	FAU	-0.134	0.025321
193	SSBP2	0.400879	0.01755461	248	CD24	0.138209	0.028542	281	RPL27	-0.13446	1.87E-09
194	SPINT1	0.40031	0.00023275	249	ODAM	0.12557	0.004247	282	UBA52	-0.13682	0.00077
195	IGKV2-24	0.399223	0.0004937	250	BGLAP	0.112763	9.18E-06	283	RPL10	-0.13705	3.15E-14
196	JCHAIN	0.390625	1.01E-05	251	SVIL	0.106956	4.17E-05	284	RPS29	-0.13804	4.38E-18
197	IGHG3	0.384059	2.19E-06	252	COL3A1	0.033749	0.046026	285	RPS6	-0.13812	6.46E-06
198	SEC11C	0.37378	0.04056081	253	COL1A2	-0.03229	0.00339	286	AEBP1	-0.13902	0.019162
199	HSF4	0.372345	0.04628472	254	COL1A1	-0.05574	9.53E-13	287	RPL13A	-0.13904	1.09E-14
200	METAP2	0.371104	0.00122611	255	ACTG1	-0.05954	0.030473	288	RPL14	-0.14011	0.006914
201	IGHA1	0.364876	5.69E-26	256	ACTB	-0.06083	0.017643	289	FN1	-0.14194	3.89E-06
202	GPC3	0.358168	0.03286435	257	RPS27	-0.06567	1.46E-11	290	RPL28	-0.14722	0.000326
203	PLEKHG4	0.355747	0.0107113	258	KRT5	-0.07848	0.000118	291	RPL32	-0.14773	2.41E-07
204	WASH5P	0.343812	0.00032504	259	RPL18	-0.08549	0.041955	292	BTF3	-0.14997	0.013162
227	FRMD4A	0.270287	3.78E-18	260	FTL	-0.09395	1.71E-05	293	TMSB4X	-0.15028	2.44E-18
228	TNFRSF21	0.267696	0.00782676	261	VIM	-0.09669	0.015105	294	PPIA	-0.15058	1.88E-10
229	S100A9	0.263898	1.89E-74	262	PFDN5	-0.11263	0.040315	295	COX4I1	-0.15059	0.041238
230	ODAPH	0.255828	7.42E-05	263	CD63	-0.11376	0.002966	296	EEF1G	-0.15252	2.66E-05
231	PDE4DIP	0.252307	0.01757775	264	MXRA8	-0.12071	0.04632	297	CALR	-0.15289	0.001465
232	SERPINB1	0.249908	0.03354334	265	CALD1	-0.12307	0.015105	298	RPL34	-0.15292	1.28E-20
233	POSTN	0.247422	7.86E-106	266	RPS14	-0.12471	8.19E-08	299	RPL5	-0.15317	2.07E-08
234	SPRR2A	0.245947	3.99E-09	267	RPS16	-0.125	1.68E-06	300	RPL15	-0.15365	2.14E-07
235	PITX2	0.235073	0.01568408	268	RPS11	-0.12502	5.46E-14	301	FBLN1	-0.15444	0.014945
236	TYMP	0.228768	0.0294623	269	RACK1	-0.12824	5.41E-06	302	LDHA	-0.15547	0.009921
237	SSR4	0.221914	0.00018482	270	LAPTM4A	-0.12937	0.005414	303	RPL39	-0.15589	4.24E-35
238	PTPRD	0.218027	0.04045423	271	COL16A1	-0.12956	0.02669	304	RPL35A	-0.15691	4.40E-09

	Symbol	Log2FC	pVal		Symbol	Log2FC	pVal		Symbol	Log2FC	pVal
305	HMGN2	-0.15763	0.01587855	338	HSPA8	-0.18174	7.81E-09	371	IFI16	-0.21398	0.038078
306	RPL8	-0.15785	7.52E-08	339	COX8A	-0.18181	0.027104	372	S100A2	-0.21429	0.000754
307	HNRNPA1	-0.15807	2.01E-06	340	RPL38	-0.18323	1.51E-10	373	RPS7	-0.21502	5.72E-11
308	HSP90AB1	-0.15883	0.00753292	341	ATP5ME	-0.18492	0.008954	374	RPS10	-0.21535	1.95E-15
309	C1R	-0.15902	0.04436527	342	RPLP0	-0.1855	7.90E-16	375	TMBIM4	-0.21589	0.001945
310	MORF4L1	-0.15908	0.00155343	343	RPL31	-0.18593	2.12E-15	376	OLFML3	-0.2181	0.039656
311	RPS5	-0.15951	9.16E-05	344	RPLP2	-0.18671	5.84E-07	377	PCOLCE	-0.21875	1.45E-08
312	UBL5	-0.15994	0.0089157	345	CLU	-0.1871	1.10E-06	378	PRCP	-0.22167	0.013494
313	NACA	-0.16023	5.19E-11	346	RPS25	-0.18936	4.91E-11	379	TCIRG1	-0.2225	0.001548
314	BGN	-0.16061	0.01430564	347	NME2	-0.19032	0.001125	380	RPL23	-0.22496	1.19E-22
315	RPL19	-0.16104	4.25E-11	348	RPL10A	-0.1908	5.64E-12	381	PMP22	-0.22542	0.044845
316	RPL30	-0.16154	4.79E-15	349	RPS13	-0.19224	3.61E-10	382	RPL11	-0.22756	5.91E-16
317	SERPINF1	-0.16268	1.94E-09	350	NAP1L1	-0.19304	0.003034	383	YBX3	-0.2286	0.018931
318	EEF1A1	-0.16398	1.33E-49	351	HSP90AA1	-0.19363	0.002097	384	C6orf48	-0.22901	0.015127
319	RPSA	-0.16489	5.11E-06	352	UBB	-0.1941	0.000167	385	RPS24	-0.23247	4.10E-35
320	CADM1	-0.16497	0.03542	353	RPL35	-0.19493	1.42E-12	386	IGFBP5	-0.23407	1.01E-12
321	LUM	-0.16603	2.03E-35	354	RPL7A	-0.19568	4.91E-11	387	RPS23	-0.23588	2.12E-52
322	RPS4X	-0.16623	6.04E-13	355	RPL13	-0.19685	5.26E-25	388	OLFML2B	-0.239	0.005816
323	GAPDH	-0.16891	1.60E-05	356	CTNNB1	-0.19688	0.000226	389	OST4	-0.24003	0.000454
324	RPS3	-0.17216	5.83E-16	357	EEF1B2	-0.19789	0.00191	390	RPS12	-0.2416	2.01E-23
325	NDUFB1	-0.17446	0.00222339	358	SERPINH1	-0.19991	6.46E-06	391	RPS3A	-0.24197	7.66E-27
326	RPL6	-0.17453	1.36E-10	359	IFITM2	-0.20129	0.01016	392	IFITM1	-0.24603	8.13E-05
327	RPS8	-0.17532	2.11E-10	360	TPT1	-0.20149	2.52E-19	393	RPL9	-0.24637	1.09E-29
328	RPL26	-0.17585	4.60E-18	361	RPL27A	-0.20357	6.00E-06	394	RPL7	-0.24879	9.84E-19
329	RPL18A	-0.17732	1.13E-05	362	ATP5F1A	-0.20536	0.000645	395	CXCL8	-0.25172	0.009969
330	RPL21	-0.17868	2.33E-10	363	NDRG1	-0.20644	0.002032	396	TGFB3	-0.25215	0.000932
331	RPS15	-0.17879	2.42E-09	364	RPL41	-0.20743	2.06E-62	397	RPS9	-0.25399	6.53E-25
332	RPS18	-0.17915	6.50E-31	365	RPL24	-0.20797	8.11E-16	398	IGFBP4	-0.2558	1.10E-06
333	RPL22	-0.17919	0.01459811	366	RPS17	-0.20815	3.77E-43	399	EGR1	-0.26062	0.033863
334	MORF4L2	-0.18	0.0004658	367	RPL12	-0.21103	2.64E-14	400	TBCA	-0.26287	0.00578
335	MRPL33	-0.1807	0.02323094	368	LGALS3BP	-0.21128	0.027726	401	S100A13	-0.26549	0.009429
336	CYR61	-0.18151	0.00971163	369	RPS20	-0.21186	2.34E-20	402	CPXM1	-0.27262	0.009051
337	TMSB10	-0.18163	1.17E-15	370	RPL37A	-0.21271	1.19E-17	403	A2M	-0.2754	1.50E-06

	Symbol	Log2FC	pVal		Symbol	Log2FC	pVal		Symbol	Log2FC	pVal
404	RPL29	-0.27591	1.47E-09	436	KRT17	-0.37727	1.02E-08	468	CA2	-0.51731	0.0133295
405	RPS2	-0.27611	4.44E-21	437	RGCC	-0.38404	0.0283	469	ITSN1	-0.5217	0.0494689
406	PRSS35	-0.27721	0.00148069	438	INTS10	-0.38531	0.014385	470	MMP13	-0.5341	5.44E-98
407	LDHB	-0.28238	1.64E-06	439	PRSS23	-0.38629	0.000122	471	IGFBP3	-0.54367	2.10E-10
408	SERPINB5	-0.28329	0.02885693	440	IGF2	-0.38716	0.033498	472	ADAM12	-0.54777	0.048098
409	RPL36A	-0.28419	5.82E-39	441	CRYAB	-0.39376	5.81E-06	473	IL6ST	-0.5502	6.63E-11
410	RPL37	-0.2871	2.77E-12	442	TNC	-0.39454	3.02E-21	474	LGALS7B	-0.56011	0.0043302
411	RARRES2	-0.28809	0.03443644	443	SLC40A1	-0.39784	3.89E-05	475	IGSF10	-0.56389	0.0003656
412	IGFBP7	-0.2959	6.01E-07	444	DOCK6	-0.39828	0.025327	476	ACP5	-0.56715	2.74E-45
413	CXCL12	-0.30079	8.87E-05	445	MDK	-0.39913	1.10E-06	477	FOSB	-0.57099	0.046194
414	CSTA	-0.3045	6.30E-05	446	PPP1R3C	-0.40151	0.041778	478	ASPG	-0.58645	0.0151051
415	NPM1	-0.3166	5.34E-13	447	COL14A1	-0.40233	0.040094	479	RELA	-0.58883	2.43E-08
416	KRT16	-0.32323	4.56E-19	448	DUT	-0.40781	1.53E-06	480	MBP	-0.60519	0.0010147
417	SLC9B2	-0.32775	0.02226288	449	MRPS27	-0.41765	0.00328	481	SPP1	-0.61261	6.55E-155
418	TIMP1	-0.33051	6.62E-13	450	SMOC2	-0.42293	0.018909	482	SLITRK6	-0.62378	0.0248711
419	SBSN	-0.33114	0.0028031	451	DSC2	-0.43261	0.017727	483	METTL22	-0.62504	0.0050867
420	RGS10	-0.33738	0.01915487	452	RNASE1	-0.44094	1.09E-09	484	ABCF1	-0.62798	3.22E-06
421	KRT6A	-0.33762	2.68E-49	453	RPL36A-HNRNPH2	-0.44101	1.20E-05	485	PLP1	-0.6377	0.0001982
422	COL11A1	-0.34044	3.22E-12	454	SELENOP	-0.44346	1.30E-41	486	MPZ	-0.63839	1.25E-09
423	KRT14	-0.34077	7.44E-75	455	SLC37A2	-0.44667	0.021789	487	PTPN12	-0.68436	3.54E-12
424	DSP	-0.34241	0.02938211	456	IBSP	-0.45963	2.27E-17	488	IGHV5-10-1	-0.68484	2.66E-05
425	MMP9	-0.34834	2.40E-18	457	PRPF19	-0.46076	0.000159	489	ARHGEF15	-0.68949	1.99E-05
426	KRT16P6	-0.34909	0.00025074	458	GNAI1	-0.46184	7.08E-06	490	LRIG3	-0.69252	1.81E-07
427	XPO1	-0.35418	0.00784465	459	OXCT1	-0.46284	0.024044	491	MT1G	-0.69832	5.81E-06
428	CTSK	-0.35443	2.70E-67	460	HEY1	-0.47131	0.042033	492	S100B	-0.71318	0.000186
429	IGLC7	-0.35496	0.01765701	461	CDK4	-0.47956	7.46E-08	493	MAP4K3	-0.72165	2.67E-05
430	DMKN	-0.35508	0.03236535	462	CFD	-0.4849	0.002146	494	TMPRSS11B	-0.73632	0.0285911
431	FOS	-0.36042	5.46E-08	463	MRPL23	-0.4864	0.000181	495	FOSL1	-0.7443	0.0347896
432	DSG3	-0.3669	0.00953547	464	PPHLN1	-0.49608	3.58E-05	496	SMIM19	-0.753	9.84E-19
433	CST3	-0.37046	2.84E-22	465	CKB	-0.49716	5.34E-10	497	HBA1	-0.75913	3.77E-13
434	RPL9P9	-0.37324	4.28E-16	466	PIK3C2A	-0.501	8.44E-07	498	IGLV3-9	-0.77982	0.0176236
435	MTIF3	-0.37454	0.01660558	467	STAT3	-0.50111	1.73E-08	499	CSNK1G3	-0.78121	0.0001452

	Symbol	Log2FC	pVal		Symbol	Log2FC	pVal		Symbol	Log2FC	pVal
500	FAH	-0.79506	0.00084059	524	DNM1	-1.14368	1.54E-95	548	TGFA	-2.22236	9.74E-97
501	PTGER4	-0.80539	0.04498582	525	ARMC8	-1.16478	2.61E-13	549	PADI4	-2.2664	0.0021579
502	AL513210.1	-0.81293	0.01864459	526	KRT76	-1.18959	7.87E-08	550	SLC24A4	-2.55269	0.0003656
503	POGZ	-0.82427	4.21E-39	527	SIK1	-1.21927	0.04338039	551	ATP13A4	-2.5765	5.46E-08
504	UIMC1	-0.8409	8.57E-05	528	CHD2	-1.28832	4.28E-43	552	BBS7	-2.64712	2.37E-199
505	PLEKHG5	-0.85093	7.27E-14	529	EPB41L1	-1.29144	6.17E-20	553	KMT2B	-2.70504	1.26E-117
506	YAF2	-0.85414	2.20E-06	530	AC012486.1	-1.334	1.12E-06	554	ELOVL6	-2.7072	2.18E-30
507	HBB	-0.85509	0	531	RAD51D	-1.33439	8.10E-07	555	CLHC1	-2.79855	1.24E-57
508	THUMPD2	-0.86974	0.0005957	532	PMEL	-1.34766	5.95E-05	556	KRT3	-2.85092	9.57E-06
509	RGS19	-0.89406	2.83E-05	533	RNF170	-1.35339	1.23E-15	557	ARL11	-2.90472	1.16E-05
510	AC127024.7	-0.93113	2.13E-30	534	EXOSC3	-1.3776	1.02E-14	558	AC092070.2	-3.01389	3.89E-47
511	KRT6C	-0.94564	1.59E-31	535	GRHL2	-1.50298	5.83E-06	559	GPR157	-3.11188	8.46E-162
512	A2ML1	-0.95068	0.00071553	536	ZNF592	-1.5083	5.71E-15	560	BOLA2P2	-3.15032	0.0041358
513	TLN2	-0.96069	5.34E-13	537	NEDD4L	-1.51581	3.43E-48	561	AC061999.1	-3.19689	2.53E-103
514	KLK4	-0.96433	0.00010355	538	NUF2	-1.54063	0.00543113	562	AK2	-3.20422	1.01E-52
515	CPNE3	-0.96559	1.43E-22	539	LZTS1	-1.55298	7.61E-10	563	DEFB124	-3.38753	7.08E-09
516	ZNF28	-1.00823	0.00801503	540	MCM3AP	-1.64187	1.04E-79	564	ANKRD20A19P	-3.47595	6.36E-05
517	KRT6B	-1.03525	1.16E-39	541	TENM4	-1.65446	1.66E-107	565	CALB1	-3.92444	4.94E-06
518	TMCC1	-1.04359	1.43E-07	542	AC020907.2	-1.7179	0.04619399	566	AC013470.2	-4.09851	9.60E-06
519	HBA2	-1.04785	1.04E-61	543	KIAA1755	-1.72553	1.81E-08	567	AC139491.5	-4.41317	0.0078434
520	KCNC4	-1.0869	0.00026779	544	AMTN	-1.82953	2.55E-47	568	C12orf42	-4.58255	5.53E-08
521	GPR171	-1.09876	0.0330849	545	EREG	-2.0269	1.91E-07	569	TAC3	-7.14812	6.07E-18
522	CCDC91	-1.11452	1.12E-21	546	CASKIN1	-2.10769	1.60E-07	570	SLC1A6	-7.66083	0.0068037
523	APOD	-1.13485	1.82E-33	547	SVIL-AS1	-2.11891	5.09E-139				

Table S3. List of significantly enriched gene ontology (GO) terms of significantly up-regulated DEGs in occlusal hypofunction samples compared with control samples.

	ID	Description	pvalue
1	GO:0006958	complement activation, classical pathway	3.94E-80
2	GO:0002455	humoral immune response mediated by circulating immunoglobulin	3.91E-77
3	GO:0006956	complement activation	3.13E-72
4	GO:0006959	humoral immune response	3.69E-70
5	GO:0016064	immunoglobulin mediated immune response	1.42E-65
6	GO:0019724	B cell mediated immunity	3.60E-65
7	GO:0002449	lymphocyte mediated immunity	3.88E-56
8	GO:0002460	adaptive immune response based on somatic recombination of immune receptors built from immunoglobulin superfamily domains	2.06E-55
9	GO:0006909	phagocytosis	8.72E-55
10	GO:0030449	regulation of complement activation	2.13E-53
11	GO:0038094	Fc-gamma receptor signaling pathway	4.80E-52
12	GO:0002920	regulation of humoral immune response	1.13E-51
13	GO:0002429	immune response-activating cell surface receptor signaling pathway	1.42E-51
14	GO:0002757	immune response-activating signal transduction	1.42E-51
15	GO:0002433	immune response-regulating cell surface receptor signaling pathway involved in phagocytosis	7.46E-51
16	GO:0038096	Fc-gamma receptor signaling pathway involved in phagocytosis	7.46E-51
17	GO:0002431	Fc receptor mediated stimulatory signaling pathway	6.45E-50
18	GO:0006910	phagocytosis, recognition	2.34E-47
19	GO:0050900	leukocyte migration	8.58E-43
20	GO:0050853	B cell receptor signaling pathway	1.56E-42
21	GO:0002377	immunoglobulin production	2.51E-42
22	GO:0050871	positive regulation of B cell activation	2.66E-42
23	GO:0038095	Fc-epsilon receptor signaling pathway	5.22E-42
24	GO:0038093	Fc receptor signaling pathway	5.39E-42
25	GO:0006911	phagocytosis, engulfment	6.15E-41
26	GO:0042742	defense response to bacterium	1.71E-40
27	GO:0050864	regulation of B cell activation	2.35E-40
28	GO:0099024	plasma membrane invagination	1.27E-39
29	GO:0010324	membrane invagination	1.52E-38
30	GO:0008037	cell recognition	5.00E-36

31	GO:0002440	production of molecular mediator of immune response	1.28E-34
32	GO:0002697	regulation of immune effector process	2.49E-34
33	GO:0006898	receptor-mediated endocytosis	4.56E-34
34	GO:0042113	B cell activation	6.73E-32
35	GO:0050851	antigen receptor-mediated signaling pathway	2.26E-30
36	GO:0051251	positive regulation of lymphocyte activation	2.14E-29
37	GO:0002696	positive regulation of leukocyte activation	2.43E-29
38	GO:0050867	positive regulation of cell activation	1.10E-28
39	GO:0051249	regulation of lymphocyte activation	5.42E-25
40	GO:0097529	myeloid leukocyte migration	5.97E-09
41	GO:0097530	granulocyte migration	1.71E-08
42	GO:1990266	neutrophil migration	2.04E-08
43	GO:0019730	antimicrobial humoral response	2.97E-08
44	GO:0071621	granulocyte chemotaxis	3.26E-08
45	GO:0018149	peptide cross-linking	4.12E-08
46	GO:0030593	neutrophil chemotaxis	5.33E-08
47	GO:0030595	leukocyte chemotaxis	1.00E-07
48	GO:0070268	cornification	1.15E-07
49	GO:0051238	sequestering of metal ion	9.74E-07
50	GO:0060326	cell chemotaxis	4.75E-06
51	GO:0030216	keratinocyte differentiation	4.94E-06
52	GO:0009913	epidermal cell differentiation	8.01E-06
53	GO:0043588	skin development	1.47E-05
54	GO:0008544	epidermis development	1.50E-05
55	GO:0031424	keratinization	1.92E-05
56	GO:0002237	response to molecule of bacterial origin	0.000294
57	GO:0070486	leukocyte aggregation	0.000355
58	GO:0002446	neutrophil mediated immunity	0.000399
59	GO:0002523	leukocyte migration involved in inflammatory response	0.000577
60	GO:0032496	response to lipopolysaccharide	0.000721
61	GO:0097067	cellular response to thyroid hormone stimulus	0.000872
62	GO:0048871	multicellular organismal homeostasis	0.00089
63	GO:0071222	cellular response to lipopolysaccharide	0.000905
64	GO:0002283	neutrophil activation involved in immune response	0.000928
65	GO:0045730	respiratory burst	0.001009
66	GO:0060263	regulation of respiratory burst	0.00105

67	GO:0048247	lymphocyte chemotaxis	0.001061
68	GO:0032755	positive regulation of interleukin-6 production	0.001103
69	GO:0042119	neutrophil activation	0.001136
70	GO:0014823	response to activity	0.001138
71	GO:0071219	cellular response to molecule of bacterial origin	0.001146

Table S4 List of significantly enriched GO terms of significantly down-regulated DEGs in occlusal hypofunction samples compared with control samples.

	ID	Description	pvalue
1	GO:0006614	SRP-dependent cotranslational protein targeting to membrane	1.41E-107
2	GO:0006613	cotranslational protein targeting to membrane	1.13E-105
3	GO:0045047	protein targeting to ER	8.28E-102
4	GO:0000184	nuclear-transcribed mRNA catabolic process, nonsense-mediated decay	5.12E-101
5	GO:0072599	establishment of protein localization to endoplasmic reticulum	3.01E-100
6	GO:0070972	protein localization to endoplasmic reticulum	4.80E-92
7	GO:0006413	translational initiation	1.33E-84
8	GO:0019083	viral transcription	1.57E-84
9	GO:0019080	viral gene expression	1.36E-81
10	GO:0000956	nuclear-transcribed mRNA catabolic process	3.27E-80
11	GO:0006612	protein targeting to membrane	1.78E-79
12	GO:0006402	mRNA catabolic process	2.96E-68
13	GO:0006401	RNA catabolic process	4.34E-65
14	GO:0090150	establishment of protein localization to membrane	1.14E-64
15	GO:0006605	protein targeting	2.81E-59
16	GO:0002181	cytoplasmic translation	1.50E-27
17	GO:0042254	ribosome biogenesis	9.82E-20
18	GO:0022613	ribonucleoprotein complex biogenesis	8.21E-17
19	GO:0042273	ribosomal large subunit biogenesis	6.46E-16
20	GO:0042255	ribosome assembly	1.70E-15
21	GO:0042274	ribosomal small subunit biogenesis	1.87E-12
22	GO:0006364	rRNA processing	3.34E-12
23	GO:0016072	rRNA metabolic process	9.11E-12
24	GO:0000027	ribosomal large subunit assembly	5.47E-10
25	GO:0030198	extracellular matrix organization	8.97E-10
26	GO:0043062	extracellular structure organization	9.48E-10
27	GO:0022618	ribonucleoprotein complex assembly	3.97E-09
28	GO:0071826	ribonucleoprotein complex subunit organization	6.52E-09
29	GO:0000028	ribosomal small subunit assembly	9.36E-09
30	GO:0034470	ncRNA processing	1.01E-08

31	GO:0046677	response to antibiotic	1.14E-08
32	GO:0070268	cornification	2.28E-08
33	GO:0034660	ncRNA metabolic process	1.42E-07
34	GO:0043588	skin development	2.19E-07
35	GO:0001503	ossification	3.60E-07
36	GO:0002576	platelet degranulation	8.37E-07
37	GO:0006417	regulation of translation	1.04E-06
38	GO:0009913	epidermal cell differentiation	1.05E-06
39	GO:0030216	keratinocyte differentiation	1.70E-06
40	GO:0034248	regulation of cellular amide metabolic process	1.98E-06
41	GO:0007565	female pregnancy	2.17E-06
42	GO:0008544	epidermis development	4.52E-06
43	GO:0034250	positive regulation of cellular amide metabolic process	5.14E-06
44	GO:0010001	glial cell differentiation	9.45E-06
45	GO:0044706	multi-multicellular organism process	1.16E-05
46	GO:1901654	response to ketone	1.18E-05
47	GO:0045104	intermediate filament cytoskeleton organization	1.22E-05
48	GO:0031424	keratinization	1.29E-05
49	GO:0032963	collagen metabolic process	1.36E-05
50	GO:0007566	embryo implantation	1.40E-05
51	GO:0045103	intermediate filament-based process	1.40E-05
52	GO:1901798	positive regulation of signal transduction by p53 class mediator	1.95E-05
53	GO:0097305	response to alcohol	2.00E-05
54	GO:0030199	collagen fibril organization	2.06E-05
55	GO:0048771	tissue remodeling	2.69E-05
56	GO:0061448	connective tissue development	2.90E-05
57	GO:0045727	positive regulation of translation	3.23E-05
58	GO:1902947	regulation of tau-protein kinase activity	3.80E-05
59	GO:0042476	odontogenesis	4.50E-05
60	GO:0045861	negative regulation of proteolysis	5.48E-05
61	GO:2001242	regulation of intrinsic apoptotic signaling pathway	6.11E-05
62	GO:0000302	response to reactive oxygen species	8.08E-05
63	GO:2000573	positive regulation of DNA biosynthetic process	8.48E-05
64	GO:0046849	bone remodeling	8.51E-05
65	GO:0051444	negative regulation of ubiquitin-protein transferase activity	9.32E-05

66	GO:0061684	chaperone-mediated autophagy	9.32E-05
67	GO:0052547	regulation of peptidase activity	0.000103
68	GO:0042542	response to hydrogen peroxide	0.000105
69	GO:0042176	regulation of protein catabolic process	0.000117
70	GO:0015669	gas transport	0.000191
71	GO:0042063	gliogenesis	0.000209
72	GO:0001649	osteoblast differentiation	0.000239
73	GO:0042552	myelination	0.00024
74	GO:0006414	translational elongation	0.000254
75	GO:0031214	biomineral tissue development	0.000259
76	GO:0110148	biomineralization	0.000259
77	GO:0007272	ensheathment of neurons	0.000269
78	GO:0008366	axon ensheathment	0.000269
79	GO:0071897	DNA biosynthetic process	0.00028
80	GO:0022617	extracellular matrix disassembly	0.000282
81	GO:0000470	maturation of LSU-rRNA	0.000288
82	GO:1903320	regulation of protein modification by small protein conjugation or removal	0.000304
83	GO:1902229	regulation of intrinsic apoptotic signaling pathway in response to DNA damage	0.000342
84	GO:0033622	integrin activation	0.000348
85	GO:1904666	regulation of ubiquitin protein ligase activity	0.000348
86	GO:2001244	positive regulation of intrinsic apoptotic signaling pathway	0.000382
87	GO:0045109	intermediate filament organization	0.000416
88	GO:0071157	negative regulation of cell cycle arrest	0.000416
89	GO:0045453	bone resorption	0.000417
90	GO:1903321	negative regulation of protein modification by small protein conjugation or removal	0.000468
91	GO:0051216	cartilage development	0.000483
92	GO:0009612	response to mechanical stimulus	0.000503
93	GO:0050821	protein stabilization	0.000522
94	GO:0006959	humoral immune response	0.000535
95	GO:0042475	odontogenesis of dentin-containing tooth	0.000537
96	GO:0045687	positive regulation of glial cell differentiation	0.000543
97	GO:0097186	amelogenesis	0.00058
98	GO:0010951	negative regulation of endopeptidase activity	0.000618

99	GO:0010038	response to metal ion	0.000635
100	GO:0032570	response to progesterone	0.000672
101	GO:0019730	antimicrobial humoral response	0.000687
102	GO:0051591	response to cAMP	0.000841
103	GO:0051054	positive regulation of DNA metabolic process	0.0009
104	GO:2000059	negative regulation of ubiquitin-dependent protein catabolic process	0.000907
105	GO:0010466	negative regulation of peptidase activity	0.000931
106	GO:0042119	neutrophil activation	0.000937
107	GO:0000054	ribosomal subunit export from nucleus	0.000974
108	GO:0033750	ribosome localization	0.000974
109	GO:0043568	positive regulation of insulin-like growth factor receptor signaling pathway	0.000974
110	GO:0045685	regulation of glial cell differentiation	0.001001
111	GO:0061844	antimicrobial humoral immune response mediated by antimicrobial peptide	0.001001
112	GO:0010718	positive regulation of epithelial to mesenchymal transition	0.001094
113	GO:0042177	negative regulation of protein catabolic process	0.001149
114	GO:0052548	regulation of endopeptidase activity	0.001172
115	GO:0071428	rRNA-containing ribonucleoprotein complex export from nucleus	0.001225
116	GO:0051438	regulation of ubiquitin-protein transferase activity	0.001308
117	GO:1902253	regulation of intrinsic apoptotic signaling pathway by p53 class mediator	0.001339
118	GO:0032964	collagen biosynthetic process	0.001426
119	GO:0038128	ERBB2 signaling pathway	0.001511
120	GO:0015671	oxygen transport	0.001513
121	GO:0035635	entry of bacterium into host cell	0.001513
122	GO:0043434	response to peptide hormone	0.001524
123	GO:2000278	regulation of DNA biosynthetic process	0.001578
124	GO:0048708	astrocyte differentiation	0.00172
125	GO:0007568	aging	0.001764
126	GO:0043312	neutrophil degranulation	0.001788
127	GO:0001654	eye development	0.00183
128	GO:0031647	regulation of protein stability	0.001846

Table S5. List of GO clusters of significantly up-regulated DEGs in occlusal hypofunction samples compared with control samples.

	ClusterNum	Term	pValue
1	1	Humoral immune response	2.36E-67
2	1	Immune response-activating cell surface receptor signaling pathway	2.60E-49
3	1	Immune response-activating signal transduction	2.60E-49
4	1	Adaptive immune response based on somatic recombination of immune receptors built from immunoglobulin superfamily domains	6.58E-53
5	1	Complement activation, classical pathway	1.01E-76
6	1	Humoral immune response mediated by circulating immunoglobulin	4.99E-74
7	1	Complement activation	2.67E-69
8	1	Immunoglobulin mediated immune response	7.27E-63
9	1	Defense response to bacterium	1.68E-38
10	1	Positive regulation of B cell activation	3.09E-40
11	1	Phagocytosis, recognition	3.33E-45
12	2	Positive regulation of leukocyte activation	1.68E-27
13	2	Positive regulation of cell activation	7.40E-27
14	2	Regulation of lymphocyte activation	3.55E-23
15	2	B cell activation	5.06E-30
16	2	Regulation of B cell activation	2.23E-38
17	2	Antigen receptor-mediated signaling pathway	1.65E-28
18	2	Positive regulation of lymphocyte activation	1.52E-27
19	2	Cell recognition	4.26E-34
20	2	B cell receptor signaling pathway	1.99E-40
21	2	Phagocytosis, engulfment	6.29E-39
22	2	Plasma membrane invagination	1.16E-37
23	2	Membrane invagination	1.34E-36
24	3	Neutrophil mediated immunity	0.017571
25	3	Neutrophil activation involved in immune response	0.03703
26	3	Neutrophil activation	0.041245
27	3	Antimicrobial humoral response	1.77E-06
28	3	Sequestering of metal ion	5.08E-05
29	3	Leukocyte aggregation	0.015923
30	3	Leukocyte migration involved in inflammatory response	0.025
31	4	Epidermis development	0.000711

32	4	Skin development	0.000709
33	4	Epidermal cell differentiation	0.000394
34	4	Keratinocyte differentiation	0.000247
35	4	Keratinization	0.000891
36	4	Cornification	6.13E-06
37	4	Peptide cross-linking	2.34E-06
38	5	Regulation of immune effector process	1.99E-32
39	5	Fc-gamma receptor signaling pathway	1.12E-49
40	5	Immune response-regulating cell surface receptor signaling pathway involved in phagocytosis	1.19E-48
41	5	Receptor-mediated endocytosis	3.53E-32
42	5	Regulation of complement activation	5.45E-51
43	5	Immunoglobulin production	3.06E-40
44	5	Production of molecular mediator of immune response	1.05E-32
45	5	Fc-epsilon receptor signaling pathway	5.73E-40
46	6	Lymphocyte mediated immunity	1.42E-53
47	6	Phagocytosis	2.48E-52
48	6	B cell mediated immunity	1.53E-62
49	6	Leukocyte migration	1.15E-40
50	6	Fc receptor signaling pathway	5.73E-40
51	6	Regulation of humoral immune response	2.40E-49
52	6	Fc-gamma receptor signaling pathway involved in phagocytosis	1.19E-48
53	6	Fc receptor mediated stimulatory signaling pathway	9.70E-48
54	7	Myeloid leukocyte migration	3.82E-07
55	7	Leukocyte chemotaxis	5.46E-06
56	7	Cell chemotaxis	0.000243
57	7	Granulocyte migration	1.07E-06
58	7	Response to molecule of bacterial origin	0.01342
59	7	Neutrophil migration	1.24E-06
60	7	Granulocyte chemotaxis	1.89E-06
61	7	Response to lipopolysaccharide	0.030714
62	7	Neutrophil chemotaxis	2.96E-06
63	7	Cellular response to lipopolysaccharide	0.036723
64	7	Cellular response to molecule of bacterial origin	0.041245
65	7	Positive regulation of interleukin-6 production	0.041245
66	7	Lymphocyte chemotaxis	0.040459

67	8	Multicellular organismal homeostasis	0.036679
68	8	Response to activity	0.041245
69	8	Respiratory burst	0.039675
70	8	Cellular response to thyroid hormone stimulus	0.036541
71	8	Regulation of respiratory burst	0.040459

Table S6. List of GO clusters of significantly down-regulated DEGs in occlusal hypofunction samples compared with control samples.

	ClusterNum	Term	pValue
1	1	Response to antibiotic	1.27E-06
2	1	Response to metal ion	0.02224
3	1	Female pregnancy	0.000183
4	1	Multi-multicellular organism process	0.000892
5	1	Response to alcohol	0.001308
6	1	Response to ketone	0.000892
7	1	Response to reactive oxygen species	0.004517
8	1	Aging	0.0489
9	1	Response to mechanical stimulus	0.018937
10	1	Response to hydrogen peroxide	0.005364
11	1	Embryo implantation	0.000951
12	1	Response to cAMP	0.028571
13	1	Response to progesterone	0.023305
14	2	Glial cell differentiation	0.000744
15	2	Gliogenesis	0.010221
16	2	Myelination	0.011412
17	2	Ensheathment of neurons	0.011951
18	2	Axon ensheathment	0.011951
19	2	Regulation of glial cell differentiation	0.031259
20	2	Astrocyte differentiation	0.048068
21	2	Positive regulation of glial cell differentiation	0.019621
22	3	Ossification	3.56E-05
23	3	Tissue remodeling	0.001695
24	3	Osteoblast differentiation	0.011412
25	3	Odontogenesis	0.002642
26	3	Biomineral tissue development	0.011828
27	3	Biomineralization	0.011828
28	3	Bone remodeling	0.004609
29	3	Odontogenesis of dentin-containing tooth	0.019579
30	3	Bone resorption	0.016254
31	3	Positive regulation of epithelial to mesenchymal transition	0.033856
32	3	Amelogenesis	0.020708

33	4	Regulation of cellular amide metabolic process	0.000172
34	4	Regulation of translation	9.60E-05
35	4	Humoral immune response	0.019579
36	4	Positive regulation of cellular amide metabolic process	0.000414
37	4	Positive regulation of translation	0.001963
38	4	Regulation of intrinsic apoptotic signaling pathway in response to DNA damage	0.014181
39	5	Extracellular matrix organization	1.24E-07
40	5	Extracellular structure organization	1.26E-07
41	5	Regulation of peptidase activity	0.005328
42	5	Regulation of endopeptidase activity	0.035632
43	5	Connective tissue development	0.001795
44	5	Negative regulation of endopeptidase activity	0.021859
45	5	Negative regulation of peptidase activity	0.030643
46	5	Cartilage development	0.018394
47	5	Collagen metabolic process	0.000951
48	5	Collagen fibril organization	0.00132
49	5	Extracellular matrix disassembly	0.012196
50	5	Collagen biosynthetic process	0.041892
51	6	Skin development	2.23E-05
52	6	Epidermis development	0.000373
53	6	Epidermal cell differentiation	9.60E-05
54	6	Keratinocyte differentiation	0.000151
55	6	Keratinization	0.000928
56	6	Cornification	2.47E-06
57	6	Intermediate filament cytoskeleton organization	0.000902
58	6	Intermediate filament-based process	0.000951
59	6	Maturation of LSU-rRNA	0.012327
60	6	Intermediate filament organization	0.016254
61	7	Eye development	0.049945
62	7	Platelet degranulation	8.06E-05
63	7	Translational elongation	0.011828
64	7	Gas transport	0.009469
65	7	Integrin activation	0.014181
66	7	Erbb2 signaling pathway	0.043298
67	7	Oxygen transport	0.043298

68	8	Response to peptide hormone	0.043298
69	8	Antimicrobial humoral response	0.023571
70	8	Antimicrobial humoral immune response mediated by antimicrobial peptide	0.031259
71	8	Ribosomal subunit export from nucleus	0.030961
72	8	Ribosome localization	0.030961
73	8	Positive regulation of insulin-like growth factor receptor signaling pathway	0.030961
74	8	Rrna-containing ribonucleoprotein complex export from nucleus	0.036918
75	8	Entry of bacterium into host cell	0.043298
76	9	Negative regulation of proteolysis	0.003167
77	9	Regulation of protein catabolic process	0.005885
78	9	Regulation of protein modification by small protein conjugation or removal	0.012864
79	9	Regulation of protein stability	0.049976
80	9	Regulation of intrinsic apoptotic signaling pathway	0.003473
81	9	Protein stabilization	0.019451
82	9	Negative regulation of protein catabolic process	0.03523
83	9	Negative regulation of protein modification by small protein conjugation or removal	0.018033
84	9	Positive regulation of intrinsic apoptotic signaling pathway	0.015393
85	9	Positive regulation of signal transduction by p53 class mediator	0.001297
86	9	Negative regulation of ubiquitin-dependent protein catabolic process	0.030226
87	9	Regulation of ubiquitin-protein transferase activity	0.039089
88	9	Negative regulation of ubiquitin-protein transferase activity	0.004894
89	9	Regulation of ubiquitin protein ligase activity	0.014181
90	9	Negative regulation of cell cycle arrest	0.016254
91	9	Regulation of intrinsic apoptotic signaling pathway by p53 class mediator	0.039661
92	10	Neutrophil activation	0.030643
93	10	Neutrophil degranulation	0.049187
94	10	Dna biosynthetic process	0.012196
95	10	Positive regulation of DNA metabolic process	0.030226
96	10	Positive regulation of DNA biosynthetic process	0.004609
97	10	Regulation of DNA biosynthetic process	0.044454

98	10	Regulation of tau-protein kinase activity	0.002271
99	10	Chaperone-mediated autophagy	0.004894
100	11	Mrna catabolic process	8.54E-66
101	11	Rna catabolic process	1.16E-62
102	11	Protein targeting	6.48E-57
103	11	Translational initiation	6.57E-82
104	11	Establishment of protein localization to membrane	2.83E-62
105	11	Nuclear-transcribed mRNA catabolic process	1.13E-77
106	11	Srp-dependent cotranslational protein targeting to membrane	4.89E-104
107	11	Cotranslational protein targeting to membrane	1.97E-102
108	11	Protein targeting to ER	9.56E-99
109	11	Nuclear-transcribed mRNA catabolic process, nonsense-mediated decay	4.43E-98
110	11	Establishment of protein localization to endoplasmic reticulum	2.09E-97
111	11	Protein localization to endoplasmic reticulum	2.78E-89
112	11	Viral transcription	6.79E-82
113	11	Viral gene expression	5.23E-79
114	11	Protein targeting to membrane	5.61E-77
115	11	Ribonucleoprotein complex biogenesis	1.58E-14
116	11	Ribosome biogenesis	2.00E-17
117	11	Cytoplasmic translation	3.25E-25
118	11	Ncrna metabolic process	1.50E-05
119	11	Ncrna processing	1.17E-06
120	11	Rrna processing	5.26E-10
121	11	Rrna metabolic process	1.37E-09
122	11	Ribonucleoprotein complex assembly	5.10E-07
123	11	Ribonucleoprotein complex subunit organization	8.08E-07
124	11	Ribosomal large subunit biogenesis	1.18E-13
125	11	Ribosome assembly	2.94E-13
126	11	Ribosomal small subunit biogenesis	3.09E-10
127	11	Ribosomal large subunit assembly	7.91E-08
128	11	Ribosomal small subunit assembly	1.12E-06
